# Polygenic Interactions With Environmental Exposures in Blood Pressure Regulation: The HUNT Study

**DOI:** 10.1161/JAHA.123.034612

**Published:** 2024-09-18

**Authors:** Karsten Øvretveit, Emma M. L. Ingeström, Michail Spitieris, Vinicius Tragante, Laurent F. Thomas, Ingelin Steinsland, Ben M. Brumpton, Daniel F. Gudbjartsson, Hilma Holm, Kari Stefansson, Ulrik Wisløff, Kristian Hveem

**Affiliations:** ^1^ HUNT Center for Molecular and Clinical Epidemiology (MCE), Department of Public Health and Nursing Norwegian University of Science and Technology (NTNU) Trondheim Norway; ^2^ Cardiac Exercise Research Group (CERG), Department of Circulation and Medical Imaging Norwegian University of Science and Technology (NTNU) Trondheim Norway; ^3^ Department of Mathematical Sciences Norwegian University of Science and Technology (NTNU) Trondheim Norway; ^4^ deCODE Genetics/Amgen Inc. Reykjavik Iceland; ^5^ Department of Clinical and Molecular Medicine Norwegian University of Science and Technology (NTNU) Trondheim Norway; ^6^ HUNT Research Centre, Department of Public Health and Nursing Norwegian University of Science and Technology (NTNU) Levanger Norway; ^7^ School of Engineering and Natural Sciences University of Iceland Reykjavik Iceland; ^8^ Faculty of Medicine University of Iceland Reykjavik Iceland; ^9^ Department of Innovation and Research, St. Olav’s Hospital Trondheim Norway

**Keywords:** blood pressure, cardiorespiratory fitness, cardiovascular disease, gene–environment interactions, polygenic risk scores, Genetics, Precision Medicine, High Blood Pressure, Hypertension, Lifestyle

## Abstract

**Background:**

The essential hypertension phenotype results from an interplay between genetic and environmental factors. The influence of lifestyle exposures such as excess adiposity, alcohol consumption, tobacco use, diet, and activity patterns on blood pressure (BP) is well established. Additionally, polygenic risk scores for BP traits are associated with clinically significant phenotypic variation. However, interactions between genetic and environmental risk factors in hypertension morbidity and mortality are poorly characterized.

**Methods and Results:**

We used genotype and phenotype data from up to 49 234 participants from the HUNT (Trøndelag Health Study) to model gene–environment interactions between genome‐wide polygenic risk scores for systolic BP and diastolic BP and 125 environmental exposures. Among the 125 environmental exposures assessed, 108 and 100 were independently associated with SBP and DBP, respectively. Of these, 12 interactions were identified for genome‐wide PRSs for systolic BP and 4 for genome‐wide polygenic risk scores for diastolic BP, 2 of which were overlapping (*P* < 2 × 10^−4^). We found evidence for gene‐dependent influence of lifestyle factors such as cardiorespiratory fitness, dietary patterns, and tobacco exposure, as well as biomarkers such as serum cholesterol, creatinine, and alkaline phosphatase on BP.

**Conclusions:**

Individuals that are genetically susceptible to high BP may be more vulnerable to common acquired risk factors for hypertension, but these effects appear to be modifiable. The gene‐dependent influence of several common acquired risk factors indicates the potential of genetic data combined with lifestyle assessments in risk stratification, and gene–environment‐informed risk modeling in the prevention and management of hypertension.

**Nonstandard Abbreviations and Acronyms**
eCRF: estimated cardiorespiratory fitness
G–E: gene–environment
HUNT: Trøndelag Health Study
PRS: polygenic risk score
RHR: resting heart rate

**Clinical Perspective**

**What Is New?**

- This paper models 250 gene–environment interactions for blood pressure traits using comprehensive polygenic risk scores and various lifestyle exposures, uncovering multiple novel interactions.

**What Are the Clinical Implications?**

- Several modifiable risk factors for hypertension may have differential effects on phenotypic expression depending on an individual's genotype.
- The inherited susceptibility to hypertension is not deterministic but influenced by common lifestyle exposures.

Blood pressure (BP) is the force distributed across arterial walls by circulating blood throughout the cardiac cycle. Its regulation within the physiological range is influenced by cardiac output, vessel characteristics, and systemic vascular resistance. In addition to being highly polygenic,^1^, ^2^ BP traits are associated with environmental factors such as diet, smoking, alcohol intake, adiposity, physical activity, psychosocial stress, and socioeconomic status.^3^, ^4^ Chronically elevated BP, or hypertension, affects over 1 billion people worldwide and is considered the leading risk factor for cardiovascular disease and chronic kidney disease.^5^, ^6^, ^7^

Although genetic risk constructs such as polygenic risk scores (PRSs) are becoming increasingly robust predictors for complex traits,^8^ they still only account for a small fraction of phenotypic variability. Moreover, a high genetic risk for hypertension is not deterministic and it is the condition itself that drives disease risk,^9^ which is expected for highly modifiable phenotypes.^10^ The single‐nucleotide polymorphism (SNP)‐based heritability for hypertension is lower than estimates from heritability analyses,^11^ but much of this discrepancy may be explained by shared familial environmental factors.^12^ Thus, although numerous genetic variants are implicated in the biology of BP regulation,^1^ the environment appears to be the main driver of phenotypic expression.

Several genetic risk variants for BP are associated with lifestyle factors. Yet, there have been few large‐scale gene–environment (G–E) interaction studies in hypertension,^13^ but the field is emerging.^14^ Recent evidence suggests that detecting any interactions at all can be challenging for this condition,^15^ which may in part be due to limitations of less comprehensive genetic risk constructs and their inability to sufficiently capture the genetic architecture of BP traits and limited or poorly characterized environmental factors. In this study, we modeled G–E interactions for BP traits using state‐of‐the‐art PRSs and a large number of environmental exposures in HUNT (the Trøndelag Health Study). Additionally, we explored the relationship between estimated cardiorespiratory fitness (eCRF) and BP in the context of genetic risk. Our aim was to assess G–E interactions between BP traits and lifestyle factors and identify potential nonpharmacological treatment targets for hypertension prevention and management.

## Methods

The HUNT data used in the present study may be obtained by applying to the HUNT Data Access Committee. For further information, visit https://www.ntnu.edu/hunt/data.

### Participants

The study sample consisted of richly phenotyped and genotyped participants from the HUNT study, a population‐based health survey that commenced in 1984 and completed its fourth survey in 2019.^16^, ^17^ Genotyped participants from the third survey (HUNT3, 2006–2008)^18^ with BP phenotype data were included in this study (Table 1). The participants were genotyped using HumanCoreExome from Illumina Inc. which reads ≈600 000 genetic variants, then subsequently enriched to ≈15 m variants using statistical imputation. The sex of the participants was obtained from the Norwegian National Registry. The study was approved by the Regional Committee for Medical and Health Research Ethics (2015/2292). All participants provided written informed consent before participation. Please see Data S1 for further information on genotyping.

**Table 1:** Clinical Characteristics of the Study Participants

	All	Women	Men
Participants (n; %female)	49 234 (55%)	26 851	22 383
Age, y	53.2±15.9	52.8±16.3	53.6±15.5
Systolic blood pressure (mm Hg)	131±19	128±20	134±17
Diastolic blood pressure (mm Hg)	73±11	71±11	76±11
Body mass index (kg/m^2^)	27.2±4.4	26.9±4.9	27.5±3.8
Waist circumference (cm)	93.6±12.3	90.4±12.7	97.4±10.5
Hip circumference (cm)	103.8±8.1	103.8±9.2	103.7±6.6
Estimated cardiorespiratory fitness (mL/kg/min)	36.1±8.2	32.6±6.5	40.8±7.9
History of antihypertensive medication use (%)	22	22	23
Daily smokers (%)	17	19	15
Serum total cholesterol (mmol/L)	5.5±1.1	5.6±1.1	5.4±1.1
Serum high‐density lipoprotein cholesterol (mmol/L)	1.3±0.4	1.5±0.4	1.2±0.3
Serum triglycerides (mmol/L)	1.6±1.0	1.5±0.8	1.9±1.2
Serum creatinine (μmol/L)	81.9±18.7	75.0±15.2	90.1±19.2

Data presented as mean±SD.

### Phenotyping

The BP of each participant in the HUNT testing cohort was obtained by trained personnel using an automated oscillometric device (Critikon Dinamap 845XT and 8100, GE Medical Systems Information Technologies, Inc.) by the stationary team and XL9301 (Johnson & Johnson Medical, Inc.) by the mobile team).^19^ Three measurements were available for each participant and the mean of the last 2 were used to calculate BP. The effects of antihypertensive medication were adjusted for by adding 15 and 10 mm Hg to the observed systolic BP (SBP) and diastolic BP (DBP), respectively.^20^ All participants completed 2 extensive questionnaires, 1 before clinical examination and 1 after. From the questionnaires and clinical measurements, 125 items were selected or derived as exposures based on phenotypic relevance (Table S1). All phenotype and exposure data were obtained from the same HUNT survey. eCRF was calculated using a nonexercise prediction model that incorporates age, waist circumference, resting heart rate, and a physical activity index to determine sex‐specific eCRF:^21^

$$\begin{array}{c} \mathrm{Men}:100.27-(0.296\times \mathrm{age})-(0.369\times \text{waist}\\ \;\text{circumference})-(0.155\times \text{resting heart rate})\\ \;+(0.226\times \text{physical activity‐index}) \end{array}$$

$$\begin{array}{c} \text{Women}:74.74-(0.247\times \mathrm{age})-(0.259\times \text{waist}\\ \;\text{circumference})-(0.114\times \text{resting heart rate})\\ \;+(0.198\times \text{physical activity‐index}) \end{array}$$

### Polygenic Risk Scores

The development of the PRSs have been described in detail elsewhere.^9^ Briefly, genome‐wide PRSs for SBP (SBP_PRS_) and DBP (DBP_PRS_) were developed using the high‐dimensional Bayesian regression approach PRS‐CS.^22^ The PRSs were trained in an independent cohort from deCODE genetics (n=81 117)^23^, ^24^ and the top performing score for each trait was identified and carried over to the HUNT cohort for G–E analyses.

### Statistical Analysis

The independent relationship between each exposure and BP traits were assessed with linear regression models and log‐transformed continuous SBP and DBP as outcomes. For each significant exposure, we then fitted models that included a multiplicative interaction term (PRS×exposure).^15^ All models were restricted to complete cases for the specific exposure and adjusted for age, age^2^, and sex. Interaction effects were identified by comparing models with and without an interaction term using the partial F‐test for categorical exposures and *t* test for binary and continuous exposures. Bonferroni correction was used for family‐wise error rate control. Interactions below the significance threshold (*P* < 2×10^−4^) were further examined with the R package “interactions,”^25^ and with splines being used when appropriate to account for nonlinearity. The effect of outliers was assessed for each model with a continuous exposure. To estimate the combined effects of all significant exposures, we compared multiple regression models with the exposures and their interactions with each PRS to a base model consisting of age, age,^2^ and sex. All analyses were performed using R (v. 4.1.3).

## Results

Up to 49 234 participants from HUNT3 were available for analysis (Table 1). Of the 125 environmental exposures assessed, 108 and 100 were independently associated with SBP (Table S2) and DBP, respectively (Table S3). Among these, 12 interactions were identified for SBP (Table S4; Figures S1) and 4 for DBP (Table S5; Figure S13–S18), 2 of which were overlapping (Table 2).

**Table 2:** Gene–Environment Interactions for Blood Pressure Traits

Exposure	Type	SBP *p* value	DBP *p* value	N SBP/DBP
Maternal smoking	Binary	2.24×10^−5^ *	3.76×10^−1^	48 452/48 754
Use of Swedish moist oral snuff (snus)	Binary	3.24×10^−6^ *	6.59×10^−7^ *	47 376/47 665
Consumption of fruit and berries	Categorical	2.56×10^−7^ *	5.02×10^−4^	49 206/49 510
Consumption of vegetables	Categorical	1.63×10^−6^ *	1.31×10^−3^	49 214/49 517
Consumption of boiled potatoes	Categorical	1.23×10^−5^ *	1.91×10^−1^	48 425/48 719
Consumption of rice/pasta	Categorical	2.99×10^−5^ *	2.20×10^−1^	46 362/46 636
Consumption of sugar‐free soda/squash	Categorical	1.59×10^−4^ *	2.75×10^−1^	45 257/45 526
Binge drinking (≥ 5 glasses or more of beer, wine, or spirits in one sitting)	Categorical	7.16×10^−7^ *	1.34×10^−1^	46 636/46 913
Marital status	Categorical	2.87×10^−11^ *	2.51×10^−5^ *	49 168/49 475
Parental divorce during childhood	Categorical	2.78×10^−5^ *	1.51×10^−2^	48 195/48 490
Estimated cardiorespiratory fitness	Continuous	4.26×10^−11^ *	3.72×10^−1^	32 302/32 306
Serum total cholesterol	Continuous	8.90×10^−9^ *	1.41×10^−1^	48 170/48 338
Serum creatinine	Continuous	2.22×10^−4^	1.76×10^−9^ *	48 856/49 035
Serum alkaline phosphatase	Continuous	1.74×10^−1^	2.82×10^−6^ *	48 821/48 999

DBP indicates diastolic blood pressure; and SBP, systolic blood pressure. The *P* value for each interaction is from the comparison of an interaction model with a base model containing the covariates sex, age, and age^2^, along with the given exposure and trait polygenic risk score.

* Interaction below the Bonferroni corrected *P* value <2 × 10^−4^. Additional details on each exposure, such as questionnaire phrasing and answer options, can be found in Table S1.

**Figure 1:**
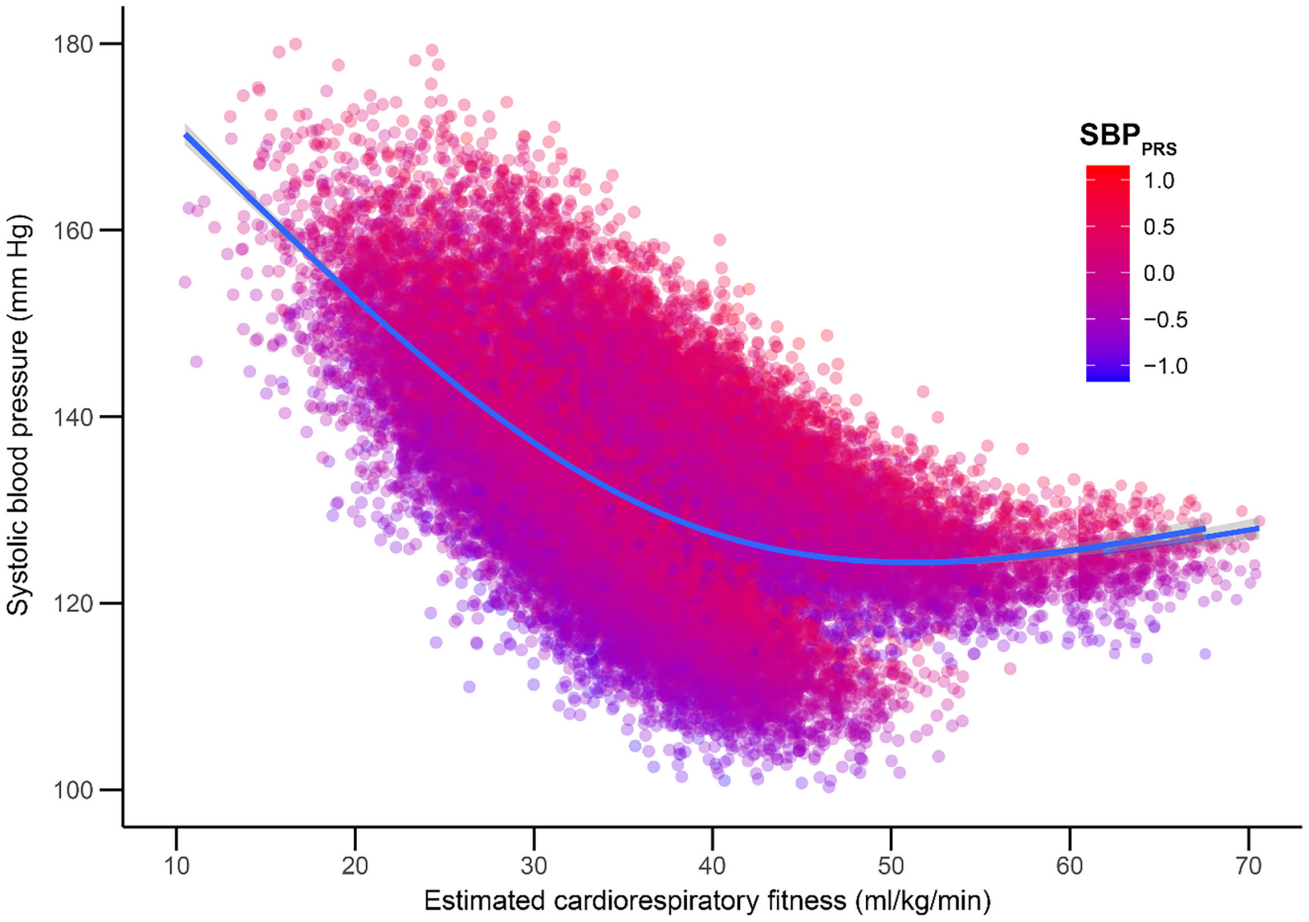
Interactions between estimated cardiorespiratory fitness and SBP_PRS_. Estimated cardiorespiratory fitness was calculated using a nonexercise prediction model.^21^ The model was adjusted for age, age^2^, and sex. SBP_PRS_ indicates polygenic risk score for systolic blood pressure.

**Figure 2:**
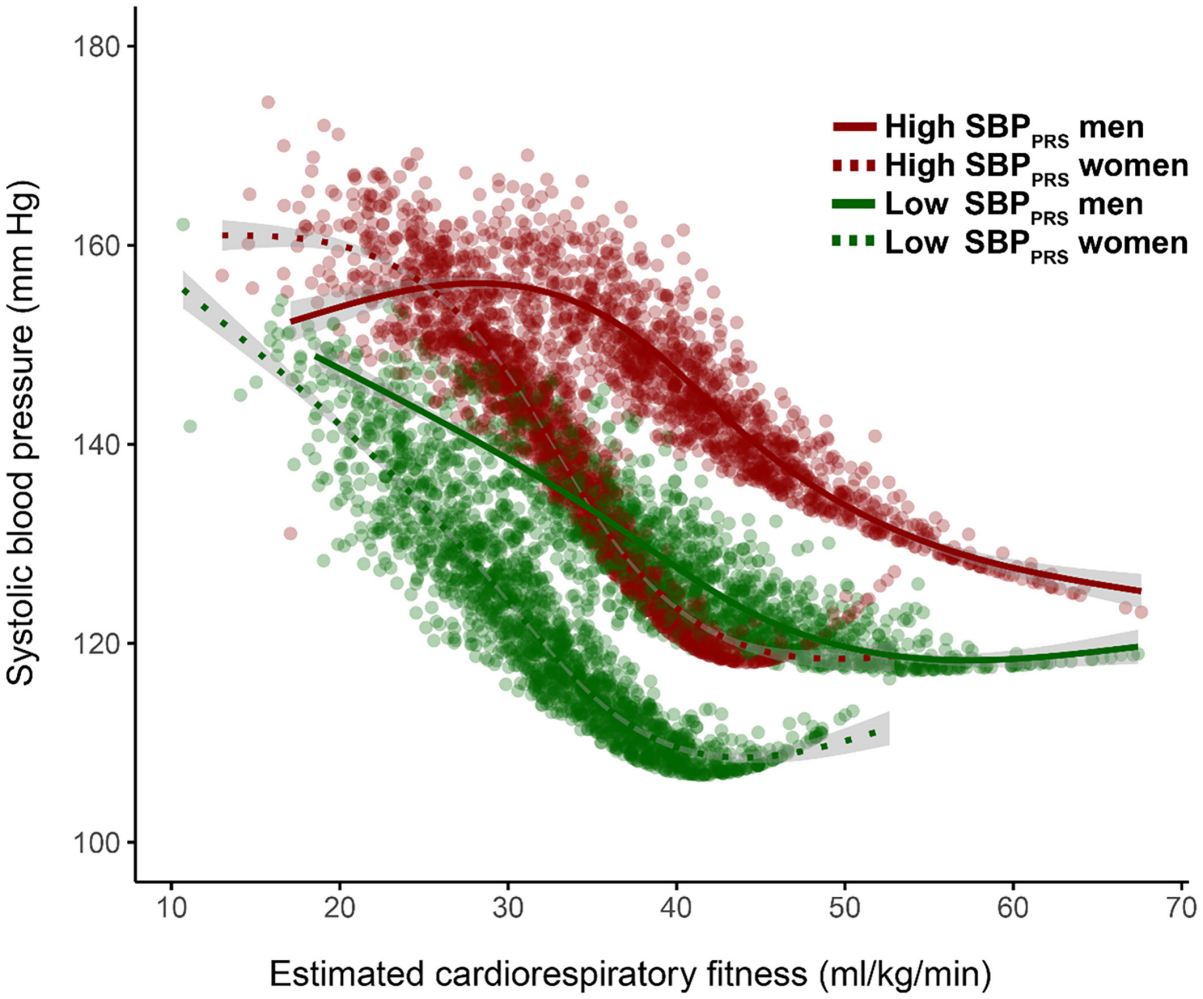
Sex‐ and risk‐stratified interactions between SBP_PRS_ and estimated cardiorespiratory fitness. Estimated cardiorespiratory fitness was calculated using a nonexercise prediction model.^21^ High SBP_PRS_ was defined as the top decile of the PRS distribution; low SBP_PRS_ was defined as the bottom decile of the PRS distribution. SBP_PRS_ indicates polygenic risk score for systolic blood pressure.

**Figure 3:**
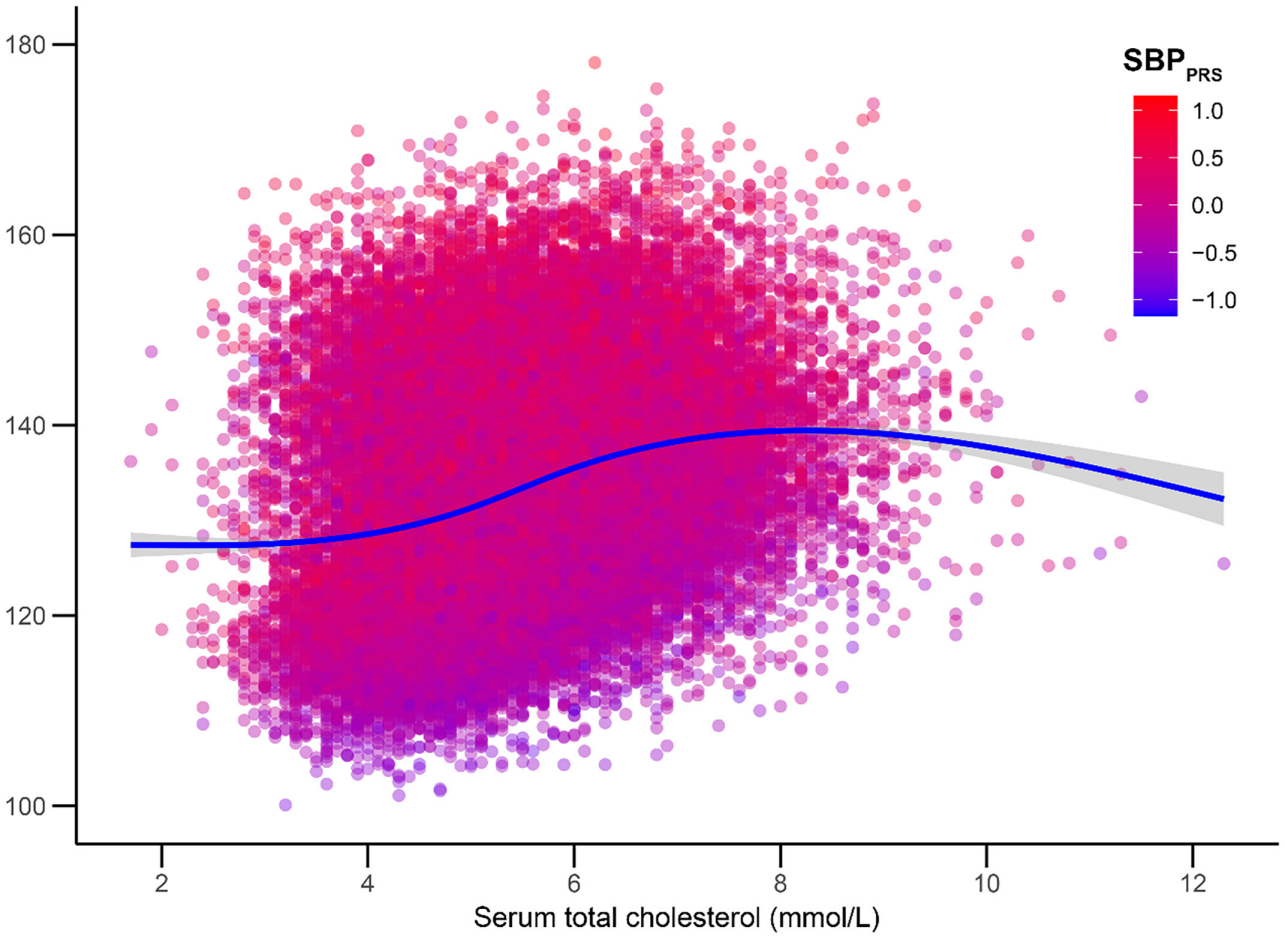
Interactions between SBP_PRS_ and serum total cholesterol. High SBP_PRS_ was defined as the top decile of the PRS distribution; low SBP_PRS_ was defined as the bottom decile of the PRS distribution. SBP_PRS_ indicates polygenic risk score for systolic blood pressure.

**Figure 4:**
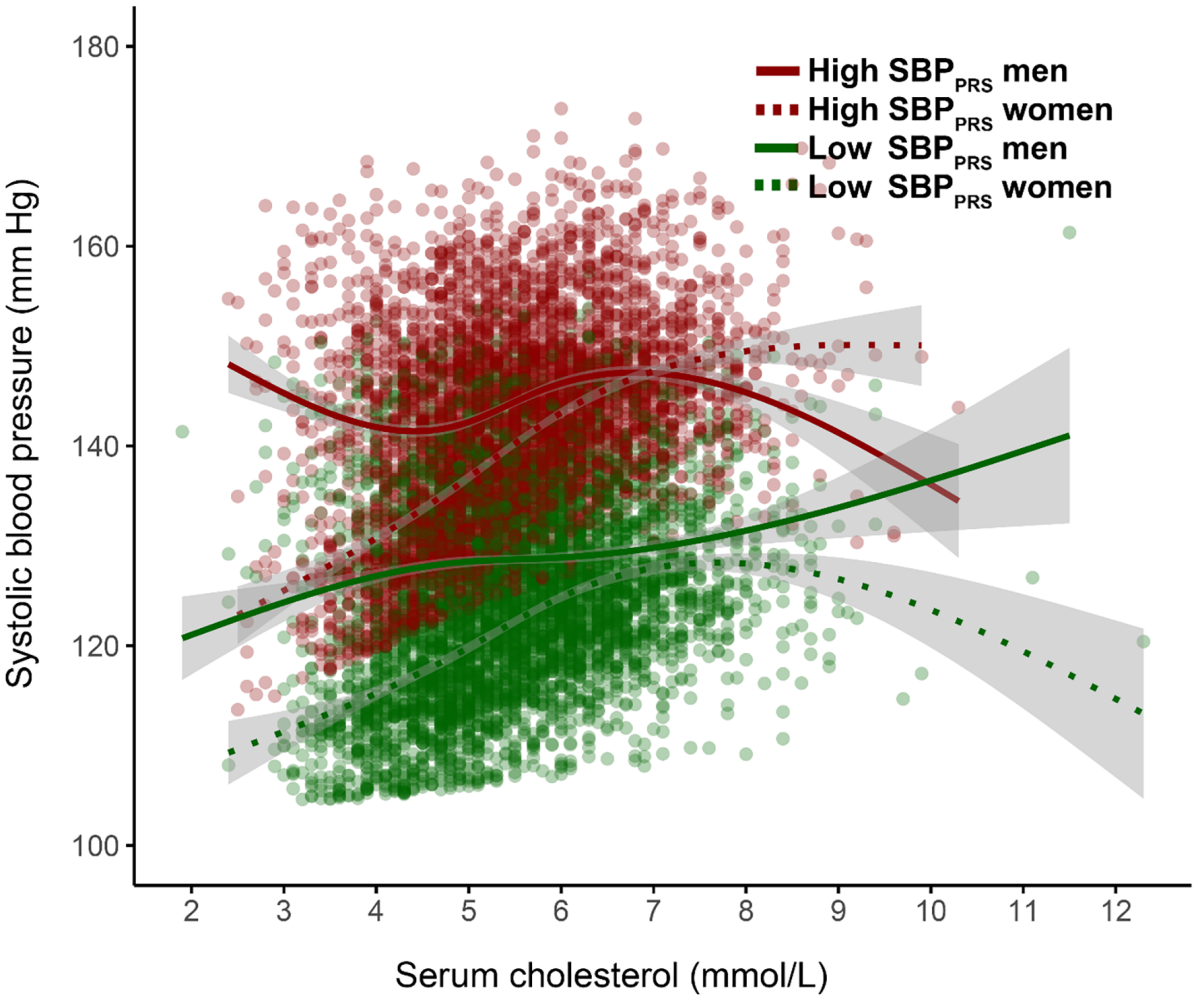
Sex‐ and risk‐stratified interactions between SBP_PRS_ and serum total cholesterol. High SBP_PRS_ was defined as the top decile of the PRS distribution; low SBP_PRS_ was defined as the bottom decile of the PRS distribution. SBP_PRS_ indicates polygenic risk score for systolic blood pressure.

We observed a beneficial interaction between eCRF and SBP across the genetic risk distribution **(**Figure 1 **)**, with a possibly larger effect in high‐risk individuals (Figure 2). In addition, we found interactions between several biomarkers and PRS, including serum cholesterol for SBP_PRS_ (Figures 3 and 4), and serum alkaline phosphatase (Figures S13 and S14) and serum creatinine (Figures S15 and S16) for DBP_PRS_.

Dietary patterns, such as the consumption of fruit, vegetables, potatoes, and rice/pasta also interacted with SBP_PRS_ (Figures S5–S8). However, given how challenging dietary exposures are to classify, caution is warranted when interpreting these results. Detrimental exposures such as maternal smoking (Figure S3) and binge drinking (Figure S10) showed somewhat counterintuitive results, with higher exposure being associated with lower SBP. The effect of Swedish moist oral snuff, or “snus,” on BP appeared to slightly be slightly modified by both SBP_PRS_ (Figure S4) and DBP_PRS_ (Figure S17). Interactions were also detected for both BP traits and marital status (Figures S11 and S18). For SBP specifically, parental marital status also appeared to interact with genetic risk (Figure S12).

## Discussion

By modeling 250 G–E interactions using PRSs for BP traits that incorporate >1 million SNPs and common lifestyle exposures, we identified 12 G–E interactions for SBP and 4 for DBP, thereby elucidating key lifestyle factors that might play an important role in BP disease pathogenesis in genetically susceptible individuals. Considering that phenotypic expression appears to override genetic susceptibility in BP‐related disease risk,^9^ modifying these exposures may mitigate poor outcomes in high‐risk individuals.

High BP is considered the most important risk factor for cardiovascular morbidity and mortality.^4^ Conversely, cardiorespiratory fitness is a major protective factor,^26^, ^27^ with no clear point of diminishing returns, particularly in those with compromised cardiovascular health, including hypertension.^28^, ^29^ By using a previously established prediction model,^21^ we were able to estimate cardiorespiratory fitness for all participants and explore interactions between this robust predictor of cardiovascular health and genetic risk factors for high BP. Notably, these estimates were within 1.1 mL/kg/min of observed values in individuals of comparable age and sex.^30^ We found an interaction between eCRF and SBP_PRS_, with a possibly greater effect in high‐risk individuals. Although this would be expected due to the law of initial value,^31^, ^32^ that is, that the magnitude of effect depends on baseline BP, it also suggests that SBP adaptability is similar, if not greater, in individuals with a high genetic susceptibility. Our observation indicates that exercise training that effectively improves cardiorespiratory fitness is a viable antihypertensive strategy in general, independent of genetic risk. This is supported by recent findings linking cardiorespiratory fitness to improved sympathetic regulation of arterial pressure and subsequently reduced BP variability in older adults.^33^

Individuals with increased genetic propensity to high SBP had higher baseline serum cholesterol concentration compared with their low‐risk counterparts Both groups had similar SBP trajectories with increases in cholesterol concentration, albeit with notable uncertainty at higher values. Even small increases in serum cholesterol concentration have been shown to affect BP regulation,^34^ supporting its role in those with increased genetic susceptibility to higher SBP. One of the mechanisms by which this relationship manifests is likely altered vessel properties due to atherosclerotic plaque formation. The atherosclerotic process starts early in life and its severity is intrinsically related to the duration of exposure to risk factors such as dyslipidemia, especially early in life.^35^ The combined effect of lifelong exposure to high cholesterol carried by low‐density lipoproteins and high SBP on cardiovascular disease risk appears to be additive and dose dependent,^36^ highlighting their interplay in cardiovascular morbidity and mortality.

The impact of diet on BP regulation is well established.^37^, ^38^ The consumption of fruit, vegetables, and dairy with low‐fat content is associated with marked reductions in BP.^39^ These effects can be further amplified by limiting sodium intake and increasing potassium intake.^40^, ^41^ We detected several interactions between dietary constituents and SBP_PRS_, suggesting that genes can influence the impact of diet on BP regulation, consistent with recent evidence.^14^ At the low end of the genetic risk spectrum, low intake of fruit and berries appeared to increase SBP. Boiled potato consumption also appeared to unfavorably affect SBP. Despite their potassium content, a high potato consumption has been shown to increase the risk of hypertension.^42^ However, because potatoes are rarely consumed by themselves, their impact on BP may be confounded by other constituents, such as sodium intake. Attempts to isolate the effects of specific foods and nutrients on hypertension should be done with caution, as it is likely the overall dietary pattern that contributes to phenotypic expression and disease risk. Thus, the diet–gene interactions observed in the present study should not be considered in isolation but rather indicative of dietary patterns being an important environmental risk factor for hypertension, with genetic propensity possibly mediating the effects of certain foods.

Alcohol consumption has a well‐established association with cardiovascular disease.^43^ The notion that alcohol may interact with certain variants to produce distinct outcomes is supported by previous research that suggests multiple BP loci interacts with alcohol consumption.^44^ We investigated interactions between PRS and several alcohol exposures and found that binge drinking, defined as the consumption of ≥5 units or more in 1 sitting, interacted with SBP_PRS_. Specifically, binge drinking was associated with lower SBP at increasing levels of genetic risk. Although some evidence suggests a higher cardiovascular disease risk for nondrinkers, this observation may be due to reverse causation or residual and unmeasured confounding.^43^ Moreover, we did not find a reliable signal for alcohol consumption using with the 5 original alcohol exposures from the data set, suggesting that the signal for the dichotomized binge drinking exposure may be spurious. Considering the several potential sources of bias, the existing evidence for a direct, linear relationship between alcohol and SBP,^45^ and the general recommendations against alcohol consumption and binge drinking in hypertension management,^3^, ^46^ further interrogation of genotype‐alcohol effects on BP is warranted.

We also observed genetic interactions for different measures of tobacco use. Previous research has revealed multiple BP loci that appear to cause interindividual differences in the BP response to smoking.^47^, ^48^ Although we did not find any interaction between genes and direct smoking exposure, the marker serum alkaline phosphatase, which is associated with smoking,^49^ interacted with DBP_PRS_. We also observed an interaction between maternal smoking and SBP_PRS_. The presence of a link between this exposure and the phenotype is consistent with the literature,^50^ but the nature of its interaction with genetic risk was challenging to interpret, with the exposure seemingly being protective among those with higher risk. A similar phenomenon was observed for the use of Swedish snus on both SBP and DBP, with current or previous use seemingly being protective. Similar to the findings on alcohol consumption, these may be spurious signals related to uncertainty in the exposed group, such as recall bias, as well as confounding factors such as socioeconomic status.

Recently, Lim et al.^15^ did not detect any G–E interactions in 2 Korean cohorts using less comprehensive BP PRSs. This supports the use of more comprehensive genetic instruments to capture the high polygenicity of BP traits. Our recently published comparison of various PRS methods for BP traits show that Bayesian approaches have greater predictive power for all BP traits compared with traditional methods with more stringent inclusion criteria and thus considerably fewer variants.^9^ These findings are consistent with the emerging literature that demonstrates the increased predictive power of genome‐wide polygenic prediction methods compared with more traditional approaches for multiple phenotypes.^22^, ^51^, ^52^, ^53^ The use of a genome‐wide PRS likely avoids some of the limitations associated with SNP‐environment interaction analysis and more accurately represents inherited risk. Moreover, because SNPs identified by exploiting G–E interaction analysis do not necessarily correspond to trait‐associated SNP,s that meet the genome‐wide significance threshold,^47^, ^54^ the inclusion of variants value above this threshold may increase the chance of detecting exposures that influence the genotype–phenotype relationship.

Disentangling the genome from the exposome for traits that are influenced by both is challenging. The G–E interactions observed in the present study indicate that genetic risk can alter the impact of several lifestyle factors that are associated with BP. Although these findings may eventually improve PRS‐based patient stratification, it is more prudent at this stage to consider them signals that warrants further investigation. To detect interactions, we created single‐exposure models and thus did not account for all potential biases, which has implications for how certain models are interpreted. Other limitations include the self‐reported data related to diet, alcohol consumption, smoking habits, and physical activity. Due to the phrasing of the Swedish snus questionnaire item, we were unable to distinguish between current and previous users, which may have affected our results. The use of antihypertensive medication was accounted for by adding constants to the observed BP,^20^ but this does not account for the number, dose, and type of medication, potentially affecting phenotypic validity. Moreover, potential interactions between treatment and other environmental exposures may increase the risk of spurious G–E signals.

Study strengths include the use of novel genome‐wide PRSs used to represent genetic risk and a richly phenotyped cohort, which allowed us to evaluate a large number of interactions. As the eCRF algorithm was developed in the same HUNT survey,^21^ it is reasonable to assume that the eCRF × PRS interaction would be similar for directly measured cardiorespiratory fitness, which is supported by the estimated values proximity to observed ones. As opposed to many other G–E study designs, we did not restrict our exposures to specific types, for example, only continuous or binary, reducing bias in our variable selection. Additionally, we used a single study cohort with standardized measurements of all participants. Although combining multiple small cohorts is common in genetic research to increase sample size, measurement variation and diverging exposure classification may make it less suitable to study interactions and can also result in a loss of power due to inflation of model error.^55^

## Conclusions

By exploring interactions between comprehensive, genome‐wide PRSs for BP and 125 environmental factors, we report 16 novel G–E interactions for BP traits. We found evidence for the protective properties of cardiorespiratory fitness and the detrimental impact of total cholesterol on SBP, with the effects of both exposures appearing to be influenced by genetic risk. Additionally, we observed gene‐dependent influence of dietary patterns and exposure to tobacco and alcohol, although these signals were challenging to interpret. Taken together, our findings suggest that genetically susceptible individuals may be more vulnerable to common acquired risk factors for hypertension pathogenesis.

## Sources of Funding

The genotyping in HUNT was financed by the National Institutes of Health (grant number NIH R35 HL135824‐03); University of Michigan; the Research Council of Norway; the Liaison Committee for Education, Research and Innovation in Central Norway; and the Joint Research Committee between St Olav's Hospital and the Faculty of Medicine and Health Sciences, Norwegian University of Science and Technology. The K. G. Jebsen Center for Genetic Epidemiology is funded by Stiftelsen Kristian Gerhard Jebsen (grant number SKGJ‐MED‐015); Faculty of Medicine and Health Sciences, Norwegian University of Science and Technology; The Liaison Committee for education, research, and innovation in Central Norway; and the Joint Research Committee between St. Olavs Hospital and the Faculty of Medicine and Health Sciences, Norwegian University of Science and Technology. This work was also partly funded by the Technology Development Fund, Iceland (project number 90580).

## Disclosures

Vinicius Tragante, Daniel F. Gudbjartsson, Hilma Holm, and Kari Stefansson declare competing financial interests as employees of deCODE genetics/Amgen Inc. The remaining authors have no disclosures to report.

## Supporting information

Data S1
Tables S1–S5
Figures S1–S18
References 56, 57
