# Supplementary material for: Polygenic Interactions With Environmental Exposures in Blood Pressure Regulation: The HUNT Study
Source: J Am Heart Assoc. 2024 Sep 18;13(19):e034612. doi: 10.1161/JAHA.123.034612 (PMC11681455; doi:10.1161/JAHA.123.034612)
Supplement: Supplementary file 1 — Data S1 Tables S1–S5 Figures S1–S18 References 56, 57 [file JAH3-13-e034612-s001.pdf]

Supplemental Material

Data S1. Supplemental Methods

Genotyping

The Trøndelag Health Study (HUNT) cohorts were genotyped using one of four different Illumina HumanCoreExome arrays (HumanCoreExome12 v1.0, HumanCoreExome12 v1.1, UM HUNT Biobank v1.0 and UM HUNT Biobank v2.0).[17] Samples from HUNT1-3 were imputed using Minimac3 (v2.0.1, http://genome.sph.umich.edu/wiki/Minimac3)[56] with default settings (2.5 Mb reference-based chunking with 500kb windows) and the HUNT-WGS customized Haplotype Reference consortium release 1.1 (HRC v1.1) for autosomal variants and HRC v1.1 for chromosome X variants.[57] More information about HUNT is available on the project's website: https://www.ntnu.edu/hunt

Interaction plots

Continuous exposures have two plots each: one with observed values for the exposure and the outcome, with splines for the main exposure effect adjusted for age, age^2, and sex; and one with predicted values of the outcome, with splines for the main exposure effect adjusted for age, age^2, sex, polygenic risk score, and the PRS × exposure interaction. Note that two of these plots are presented in the main text. Binary and categorical exposures are plotted with observed values for the outcome and the environmental exposure, with splines for the main PRS effect adjusted for age, age^2, sex, the exposure, and the PRS × exposure interaction.

Table S1. Clinical measurements and questionnaire items that were used as exposure variables

**Anthropometry**	
	Height
	Body mass
	Waist circumference
	Hip circumference
	Body mass index
**Weight reduction**	
	Are you satisfied with your weight now?
	Have you tried to diet in the last 10 years?
**Injuries and diseases**	
	Do you have pain in your neck/shoulders or lower back today?
	Do you have physical pain now that has lasted more than 6 months?
	How strong has your physical pain been during the last 4 weeks?
	How is your health at the moment?
	Mental health problems you sought help for (ever)
	Mental health problems you sought help for (age)
	CONOR_MHI average score
	Has anyone at any time in your life tried to oppress, degrade or humiliate you over an extended period of time?
**Blood markers**	
	Serum total cholesterol
	Serum HDL cholesterol
	Serum triglycerides
	Serum non-fasting glucose
	Serum creatinine
	Serum calcium
	Serum alkaline phosphatase
	Serum alanine aminotransferase
	Serum phosphate
	Thyroid-Stimulating Hormone
**Religion**	
	Which life philosophy is most like yours?
	When something bad happens in my life, I think that it happened for a purpose.
	I seek God's help when I need strength and solace.

**Alcohol**	
	About how often in the last 12 months did you drink alcohol? (Do not include low-alcohol beer)
	Did you drink alcohol during the last 4 weeks?
	Total alcohol units per week
	Total quantity in gram of pure alcohol per week
	How often do you drink 5 glasses or more of beer, wine or spirits in one sitting?
**Smoking and snus**	
	Did any of the adults where you grew up smoke indoors?
	Did your mother smoke when you were growing up?
	No, I have never smoked
	No, I have quit smoking
	Yes, cigarettes occasionally (parties/vacation, not daily)
	Yes, cigars/cigarillos/pipe occasionally
	Yes, cigarettes daily
	Yes, cigars/cigarillos/pipe daily
	Smoking status
	How many cigarettes do/did you usually smoke daily?
	How old were you when you started smoking daily?
	If you previously smoked daily; how old were you when you quit smoking?
	If you previously smoked, how long has it been since you stopped?
	How many cigarettes do/did you usually smoke in a month?
	Estimated number of years with daily smoking
	Estimated number of pack-years
	Do you use, or have you used snus?
	If Yes [using snus]: How old were you when you began using snus/chewing tobacco?
	If yes [using snus]: How many portions snus do/did you use a month?
**Location**	
	Municipality geography where participated
	Municipality urbanity where participated
**Physical activity**	
	How often do you exercise?
	How hard do you exercise? (average)
	How long do you exercise each time? (average)
	Do you have at least 30 minutes of physical activity daily at work or in your leisure time?
	About how many hours do you sit during an average day? (include work hours and leisure time)
	How much of your leisure time have you been physically active in the last year? Low intensity
	How much of your leisure time have you been physically active in the last year? High intensity

**Nutrition**
Fruits and berries (frequency)
Vegetables (frequency)
Chocolate/candy (frequency)
Boiled potatoes (frequency)
Pasta/rice (frequency)
Sausages/hamburgers (frequency)
High-fat fish (salmon, trout, herring, mackerel, haddock on bread or for dinner)
Cod-liver oil (supplement)
Omega 3 capsules (supplement)
Vitamins and/or minerals (supplement)
How many glasses of the following types of beverage do you usually drink? Water, farris, etc.
How many glasses of the following types of beverage do you usually drink? Whole milk (sweet/sour).
How many glasses of the following types of beverage do you usually drink? Other milk (sweet/sour)?
How many glasses of the following types of beverage do you usually drink? Soda/juice with sugar?
How many glasses of the following types of beverage do you usually drink? Soda/juice without sugar?
How many glasses of the following types of beverage do you usually drink? Juice or nectar.
How many cups of coffee/tea do you drink daily? Boiled coffee.
How many cups of coffee/tea do you drink daily? Other type of coffee.
How many cups of coffee/tea do you drink daily? Tea.
How many cups of coffee do you drink in the evening (after 6pm)?
How many slices of bread do you usually eat (set one tag for each type of bread)? White bread
How many slices of bread do you usually eat (set one tag for each type of bread)? Semi wholegrain bread
How many slices of bread do you usually eat (set one tag for each type of bread)? Wholegrain bread
How often do you usually eat these meals? Breakfast.
How often do you usually eat these meals? Lunch.
How often do you usually eat these meals? Warm dinner.
How often do you usually eat these meals? Supper/evening snack.
How often do you usually eat these meals? Other meals.
How often do you usually eat these meals? Midnight snack (24.00 - 06.00)
What kind of fat is usually used in your household? (on bread)
What kind of fat is usually used in your household? (in cooking)
**Sleep**
How often in the last 3 months have you had difficulty falling asleep at night?
How often in the last 3 months have you woken up repeatedly during the night?
How often in the last 3 months have you woken too early and couldn’t get back to sleep?
How often in the last 3 months have you felt sleepy during the day?
How many times do you get up during the night to urinate?
If you get up during the night to urinate, is this a problem for you?

Work
Do you have a job?
Do you have full-time work or part-time work?
Do you usually work more than 40 hours a week?
If you work part-time, what percent do you work?
Are you a salaried employee or a self-employed person?
For your main occupation, in which business sector do you work in?
Do you work shifts, at night or are on call?
Is your work so physically demanding that you are often physically worn out after a long day's work?
Are you at work exposed to noise?
Are you at work exposed to dust and smoke?
Are you at work exposed to harmful gases?
Are you at work exposed to solvents?
If you have had paid or unpaid employment, how would you describe your job?
There is a good collegiality at work.
My co-workers are there for me (support me).
I get along well with my co-workers.
Are you bullied/harassed at work?
Does your job require you to work very fast?
Does your job require you to work very hard?
Does your job require a great deal of work effort?
Does your job require creativity?
Do you have the possibility to decide for yourself how to carry out your work?
Do you have the possibility to decide for yourself what should be done in your work?
**Marriage**
Marital status
Did your parents leave each other, or get a divorce, when you were a child?
**Leisure time**
How many hours do you watch TV/video/DVD daily?

Further details about number of respondents and questionnaire alternatives can be found online at: https://hunt-db.medisin.ntnu.no/hunt-db/

Table S2. Exposure variables that were significantly associated with systolic blood pressure

Exposure	R^2	*p*-value	N
How many hours do you watch TV/video/DVD daily?	0.2473	4.22E-170	39132
Do you have at least 30 minutes of physical activity daily at work or in your leisure time?	0.2421	2.93E-29	49187
About how often in the last 12 months did you drink alcohol? (Do not include low-alcohol beer)	0.2418	2.43E-175	47900
Did you drink alcohol during the last 4 weeks?	0.2428	5.62E-134	47885
How often do you drink 5 glasses or more of beer, wine or spirits in one sitting?	0.2409	6.58E-162	46636
Body mass index	0.2844	<2.2e–16	49046
Did your parents leave each other, or get a divorce, when you were a child?	0.2431	3.44E-131	48195
How many cups of coffee/tea do you drink daily? Boiled coffee.	0.2636	2.39E-104	25078
How many cups of coffee do you drink in the evening (after 6pm)?	0.2419	8.43E-05	46918
How many cups of coffee/tea do you drink daily? Other type of coffee.	0.2422	6.83E-38	37258
How many glasses of the following types of beverage do you usually drink? Juice or nectar.	0.2417	1.76E-85	46894
How many glasses of the following types of beverage do you usually drink? Soda/squashes with sugar?	0.2420	1.26E-70	45698
How many glasses of the following types of beverage do you usually drink? Soda/squashes without sugar?	0.2450	2.56E-73	45257
How many glasses of the following types of beverage do you usually drink? Whole milk (sweet/sour)?	0.2423	6.36E-95	44029
How many glasses of the following types of beverage do you usually drink? Other milk (sweet/sour)?	0.2445	1.50E-21	46051
How many cups of coffee/tea do you drink daily? Tea.	0.2685	1.94E-22	26694
How many glasses of the following types of beverage do you usually drink? Water, farris, etc.	0.2422	4.99E-24	47819
Estimated cardiorespiratory fitness	0.2787	<2.2e–16	32302
How long do you exercise each time? (average)	0.2461	1.85E-68	37436
How often do you exercise?	0.2426	3.08E-50	48364
How much of your leisure time have you been physically active in the last year? High intensity	0.2515	1.47E-117	26968
How hard do you exercise? (average)	0.2458	1.62E-272	37251
How much of your leisure time have you been physically active in the last year? Low intensity	0.2491	6.98E-65	32644
What kind of fat is usually used in your household? (on bread)	0.2433	3.28E-29	39547
What kind of fat is usually used in your household? (in cooking)	0.2455	1.21E-91	38880
Chocolate/candy (frequency)	0.2417	<2.2e–16	46645

High-fat fish (salmon, trout, herring, mackerel, haddock on bread or for dinner)	0.2425	<2.2e–16	47974
Fruits and berries (frequency)	0.2421	1.86E-20	49206
Boiled potatoes (frequency)	0.2428	<2.2e–16	48425
Pasta/rice (frequency)	0.2424	<2.2e–16	46362
Sausages/hamburgers (frequency)	0.2400	3.65E-06	46822
Cod-liver oil (supplement)	0.2457	2.99E-126	41266
Omega 3 capsules (supplement)	0.2448	2.47E-232	44909
Vitamins and/or minerals (supplement)	0.2423	3.82E-67	44306
Vegetables (frequency)	0.2420	1.28E-41	49214
How is your health at the moment?	0.2448	<2.2e–16	47743
Height	0.2431	2.35E-14	49071
Hip circumference	0.2657	<2.2e–16	49011
Has anyone at any time in your life tried to oppress, degrade or humiliate you over an extended period of time?	0.2450	5.65E-75	45240
How often in the last 3 months have you woken too early and couldn't get back to sleep?	0.2424	3.97E-126	39173
How often in the last 3 months have you woken up repeatedly during the night?	0.2427	2.19E-75	39236
Marital status	0.2431	<2.2e–16	49168
How often do you usually eat these meals? Breakfast.	0.2438	5.75E-64	39618
How often do you usually eat these meals? Warm dinner.	0.2439	2.47E-92	39500
How often do you usually eat these meals? Lunch.	0.2439	3.38E-198	37620
How often do you usually eat these meals? Midnight snack (24.00 - 06.00)	0.2450	2.01E-10	34729
How often do you usually eat these meals? Other meals.	0.2423	8.83E-127	35090
How often do you usually eat these meals? Supper/evening snack.	0.2457	2.10E-174	38483
CONOR_MHI average score	0.2404	0.00011	45525
Mental health problems you sought help for (age)	0.2244	6.05E-142	6769
Mental health problems you sought help for (ever)	0.2438	9.80E-19	47486
How strong has your physical pain been during the last 4 weeks?	0.2422	1.46E-48	45516
Do you have physical pain now that has lasted more than 6 months?	0.2426	2.37E-52	47483
Municipality geography where participated	0.2427	5.17E-10	49022
Municipality urbanity where participated	0.2436	8.67E-12	49022
Which life philosophy is most like yours?	0.2455	2.70E-107	38759

When something bad happens in my life, I think that it happened for a purpose.	0.2455	1.43E-42	39099
I seek God's help when I need strength and solace.	0.2455	1.20E-78	38919
Serum alanine aminotransferase	0.2518	4.83E-198	48548
Serum alkaline phosphotase	0.2494	4.24e-314	48821
Serum calcium	0.2565	1.77E-191	48820
Serum total cholesterol	0.2477	<2.2e–16	48170
Serum creatinine	0.2424	<2.2e–16	48856
Serum non-fasting glucose	0.2499	<2.2e–16	48170
Serum HDL cholesterol	0.2435	3.30E-35	48169
How many slices of bread do you usually eat (set one tag for each type of bread)? Semi wholegrain bread	0.2506	5.23E-46	27722
Serum phosphate	0.2472	1.61e-319	48807
Serum triglycerides	0.2593	<2.2e–16	48856
Thyroid-Stimulating Hormone	0.2431	2.50E-31	48009
How often in the last 3 months have you felt sleepy during the day?	0.2434	1.11E-156	39186
Yes, cigars/cigarillos/pipe daily	0.2420	6.51E-11	49234
Yes, cigars/cigarillos/pipe occasionally	0.2420	5.02E-13	49234
Yes, cigarettes daily	0.2428	4.40E-53	49234
How many cigarettes do/did you usually smoke daily?	0.2063	6.37E-45	19928
How many cigarettes do/did you usually smoke in a month?	0.2313	1.30E-10	4474
Yes, cigarettes occasionally (parties/vacation, not daily)	0.2420	1.69E-27	49234
How old were you when you started smoking daily?	0.2033	7.91E-91	22747
If you previously smoked daily; how old were you when you quit smoking?	0.2014	1.09E-195	12931
If you previously smoked, how long has it been since you stopped?	0.2012	1.09E-154	12909
Estimated number of years with daily smoking	0.2096	1.40E-202	20844
Did your mother smoke when you were growing up?	0.2425	<2.2e–16	48452
No, I have never smoked	0.2426	6.90E-13	49234
Estimated number of pack-years	0.2174	7.04E-239	22575
No, I have quit smoking	0.2420	2.55E-99	49234
Smoking status	0.2436	7.00E-132	47884
If Yes [using snus]: How old were you when you began using snus/chewing tobacco?	0.2078	1.65E-99	6815

If yes [using snus]: How many portions snus do/did you use a month?	0.2075	9.89E-05	6764
Do you use, or have you used snus?	0.2408	2.03E-15	47376
How many times do you get up during the night to urinate?	0.2200	<2.2e–16	36710
Waist circumference	0.2718	<2.2e–16	49006
Body mass	0.2755	<2.2e–16	49058
Have you tried to diet in the last 10 years?	0.2462	4.20E-21	48337
Are you satisfied with your weight now?	0.2578	2.43E-77	48403
How many slices of bread do you usually eat (set one tag for each type of bread)? White bread	0.2486	9.95E-16	23062
How many slices of bread do you usually eat (set one tag for each type of bread)? Wholegrain bread	0.2461	2.63E-20	34253
Are you a salaried employee or a self-employed person?	0.1879	9.59E-31	31489
Do you have the possibility to decide for yourself how to carry out your work?	0.1788	1.02E-06	26783
Do you have the possibility to decide for yourself what should be done in your work?	0.1785	1.84E-16	26771
Do you have a job?	0.2436	<2.2e–16	49208
Is your work so physically demanding that you are often physically worn out after a long day's work?	0.2061	3.46E-08	29514
Hvis du har deltidsstilling; hvor stor stillingsandel har du?	0.2033	1.21E-17	9944
Does your job require you to work very fast?	0.1786	2.50E-08	26654
Does your job require creativity?	0.1796	1.13E-08	26736
For your main occupation, in which business sector do you work in?	0.2405	2.82E-261	45095
Do you work shifts, at night or are on call?	0.1878	5.46E-51	31668
Do you usually work more than 40 hours a week?	0.1719	1.19E-06	21501
Do you have full-time work or part-time work?	0.1876	5.72E-67	31755
If you have had paid or unpaid employment, how would you describe your job?	0.2140	1.73E-73	35821

The *p*-value was derived from a partial F-test for categorical exposures and *t*-test for binary and continuous exposures comparing the exposure model to a base model with age, age^2, and sex.

Table S3. Exposure variables that were significantly associated with diastolic blood pressure

Exposure	R^2	*p*-value	N
How many hours do you watch TV/video/DVD daily?	0.1810	1.69E-50	39239
Do you have at least 30 minutes of physical activity daily at work or in your leisure time?	0.1858	3.08E-22	49484
About how many hours do you sit during an average day? (include work hours and leisure time)	0.1984	2.26E-15	42759
About how often in the last 12 months did you drink alcohol? (Do not include low-alcohol beer)	0.1882	3.75E-99	48185
How often do you drink 5 glasses or more of beer, wine or spirits in one sitting?	0.1910	9.64E-22	46913
Total quantity in gram of pure alcohol per week	0.1926	1.82E-44	46009
Total alcohol units per week	0.1925	4.44E-42	46009
Body mass index	0.2142	<2.2e–16	49229
Did your parents leave each other, or get a divorce, when you were a child?	0.1866	3.98E-96	48490
How many cups of coffee/tea do you drink daily? Boiled coffee.	0.2208	3.30E-104	25238
How many cups of coffee do you drink in the evening (after 6pm)?	0.1868	7.98E-36	47197
How many cups of coffee/tea do you drink daily? Other type of coffee.	0.1892	2.00E-127	37462
How many glasses of the following types of beverage do you usually drink? Juice or nectar.	0.1902	4.71E-54	47174
How many glasses of the following types of beverage do you usually drink? Soda/squashes with sugar?	0.1917	2.38E-30	45968
How many glasses of the following types of beverage do you usually drink? Soda/squashes without sugar?	0.1954	1.77E-25	45526
How many glasses of the following types of beverage do you usually drink? Whole milk (sweet/sour)?	0.1947	1.32E-29	44295
How many cups of coffee/tea do you drink daily? Tea.	0.2035	1.21E-22	26861
How many glasses of the following types of beverage do you usually drink? Water, farris, etc.	0.1882	9.33E-24	48113
Estimated cardiorespiratory fitness	0.2345	<2.2e–16	32306
How long do you exercise each time? (average)	0.1923	4.18E-21	37644
How often do you exercise?	0.1882	8.45E-25	48659
How much of your leisure time have you been physically active in the last year? High intensity	0.2012	1.20E-24	27030
How hard do you exercise? (average)	0.1917	7.70E-89	37459
How much of your leisure time have you been physically active in the last year? Low intensity	0.1788	3.53E-36	32738
What kind of fat is usually used in your household? (on bread)	0.1756	2.87E-08	39654
What kind of fat is usually used in your household? (in cooking)	0.1770	1.64E-32	38980

Chocolate/candy (frequency)	0.1913	6.90E-167	46919
High-fat fish (salmon, trout, herring, mackerel, haddock on bread or for dinner)	0.1876	5.98E-140	48265
Fruits and berries (frequency)	0.1862	6.64E-10	49510
Boiled potatoes (frequency)	0.1867	<2.2e–16	48719
Pasta/rice (frequency)	0.1923	<2.2e–16	46636
Cod-liver oil (supplement)	0.1974	1.57E-27	41512
Omega 3 capsules (supplement)	0.1924	2.31E-59	45172
Vitamins and/or minerals (supplement)	0.1903	2.00E-35	44570
Vegetables (frequency)	0.1858	2.84E-20	49517
How is your health at the moment?	0.1898	2.20E-195	48034
Height	0.1862	3.80E-143	49255
Hip circumference	0.1983	1.94E-243	49196
Has anyone at any time in your life tried to oppress, degrade or humiliate you over an extended period of time?	0.1894	6.65E-22	45517
How often in the last 3 months have you had difficulty falling asleep at night?	0.1771	8.15E-09	39415
How often in the last 3 months have you woken too early and couldn't get back to sleep?	0.1780	8.50E-69	39276
How often in the last 3 months have you woken up repeatedly during the night?	0.1779	6.45E-27	39341
Marital status	0.1860	<2.2e–16	49475
How often do you usually eat these meals? Breakfast.	0.1774	8.91E-07	39724
How often do you usually eat these meals? Warm dinner.	0.1762	2.71E-15	39604
How often do you usually eat these meals? Lunch.	0.1816	1.42E-86	37712
How often do you usually eat these meals? Midnight snack (24.00 - 06.00)	0.1837	2.04E-05	34812
How often do you usually eat these meals? Other meals.	0.1827	1.38E-83	35174
How often do you usually eat these meals? Supper/evening snack.	0.1786	1.58E-26	38584
Mental health problems you sought help for	0.1622	1.70E-69	6810
How strong has your physical pain been during the last 4 weeks?	0.1901	2.10E-44	45789
Have you tried to diet in the last 10 years?	0.1883	7.06E-40	47773
Municipality geography where participated	0.1853	1.63E-22	49248
Municipality urbanity where participated	0.1867	3.46E-29	49248
Which life philosophy is most like yours?	0.1785	4.28E-21	38866
When something bad happens in my life, I think that it happened for a purpose.	0.1793	6.28E-07	39203

Serum alanine aminotransferase	0.1913	6.46E-298	48722
Serum alkaline phosphotase	0.1891	7.48E-135	48999
Serum calcium	0.1922	1.47E-90	48998
Serum total cholesterol	0.1895	<2.2e–16	48338
Serum creatinine	0.1853	2.93E-298	49035
Serum non-fasting glucose	0.1859	7.15E-208	48338
Serum HDL cholesterol	0.1864	1.12E-114	48337
How many slices of bread do you usually eat (set one tag for each type of bread)? Semi wholegrain bread	0.1942	9.32E-36	27791
Serum phosphate	0.1873	1.90E-243	48985
Serum triglycerides	0.1998	<2.2e–16	49035
Serum calcium	0.1849	4.82E-24	48177
How often in the last 3 months have you felt sleepy during the day?	0.1775	8.01E-70	39288
Yes, cigars/cigarillos/pipe daily	0.1855	5.09E-06	86569
Yes, cigars/cigarillos/pipe occasionally	0.1855	4.97E-12	86569
Yes, cigarettes daily	0.1857	7.15E-08	86569
How many cigarettes do/did you usually smoke daily?	0.1653	6.72E-78	20046
How many cigarettes do/did you usually smoke in a month?	0.2398	4.33E-12	4491
Yes, cigarettes occasionally (parties/vacation, not daily)	0.1855	6.89E-21	86569
How old were you when you started smoking daily?	0.1590	1.51E-07	22888
If you previously smoked daily; how old were you when you quit smoking?	0.1662	4.71E-56	13008
If you previously smoked, how long has it been since you stopped?	0.1660	7.58E-53	12984
Estimated number of years with daily smoking	0.1640	7.95E-90	20967
Did any of the adults where you grew up smoke indoors?	0.1863	6.98E-47	48793
Did your mother smoke when you were growing up?	0.1858	4.86E-108	48754
No, I have never smoked	0.1856	9.05E-38	86569
Estimated number of pack-years	0.1871	2.62E-198	22695
No, I have quit smoking	0.1855	3.16E-94	86569
If Yes [using snus]: How old were you when you began using snus/chewing tobacco?	0.2826	1.07E-99	6854
How many times do you get up during the night to urinate?	0.1308	3.78E-97	36810
Waist circumference	0.2093	<2.2e–16	49193

Body mass	0.2111	<2.2e–16	49241
Are you satisfied with your weight now?	0.2041	5.23E-174	48701
How many slices of bread do you usually eat (set one tag for each type of bread)? White bread	0.1977	3.81E-07	23119
How many slices of bread do you usually eat (set one tag for each type of bread)? Wholegrain bread	0.1798	2.60E-19	34345
Are you a salaried employee or a self-employed person?	0.2067	5.34E-32	31533
Do you have the possibility to decide for yourself how to carry out your work?	0.1571	7.69E-11	26823
Do you have the possibility to decide for yourself what should be done in your work?	0.1569	1.60E-18	26811
Do you have a job?	0.1858	3.77E-123	49351
Does your job require you to work very fast?	0.1559	4.24E-05	26694
For your main occupation, in which business sector do you work in?	0.1867	8.45E-286	45209
Do you work shifts, at night or are on call?	0.2061	8.43E-40	31712
Do you usually work more than 40 hours a week?	0.2141	1.06E-11	21536
Do you have full-time work or part-time work?	0.2063	8.46E-113	31804
If you have had paid or unpaid employment, how would you describe your job?	0.2126	1.32E-73	35994

The *p*-value was derived from a partial F-test for categorical exposures and *t*-test for binary and continuous exposures comparing the exposure model to a base model with age, age^2, and sex.

Table S4. Interaction models for systolic blood pressure

Did your mother smoke when you were growing up? (SmoMothCh)	β estimate	95% CI	P-value
(Intercept)	4.680446	4.669957. 4.690935	<2.2e–16
Age	0.001929	0.001522. 0.002337	1.79E-20
Age^2	2.40E-05	2e-05. 2.7e-05	6.18E-34
Sex	0.044025	0.04178. 0.046271	2.92E-318
SmoMothCh.NT3BLQ1Yes	-0.002655	-0.005295. -1.4e-05	0.048766
SYSPRS	0.138884	0.134086. 0.143683	<2.2e–16
SmoMothCh.NT3BLQ1Yes:SYSPRS	-0.018855	-0.027571. -0.010139	2.24E-05

Do you use, or have you used snus? (SnuEvEd)	β estimate	95% CI	P-value
(Intercept)	4.679403	4.668807. 4.689999	<2.2e–16
Age	0.001858	0.001444. 0.002272	1.40E-18
Age^2	2.50E-05	2.1e-05. 2.8e-05	1.03E-35
Sex	0.044037	0.041629. 0.046445	1.14E-277
SnuEvEd.NT3BLQ1Yes	0.004996	0.001564. 0.008429	0.004329
SYSPRS	0.137517	0.133116. 0.141918	<2.2e–16
SnuEvEd.NT3BLQ1Yes:SYSPRS	-0.026493	-0.037647. -0.015339	3.24E-06

Consumption frequency of fruit and berries (FoFruF)	β estimate	95% CI	P-value
(Intercept)	4.681899	4.670804. 4.692993	<2.2e–16
Age	0.002156	0.001746. 0.002566	6.71E-25
Age^2	2.20E-05	1.8e-05. 2.6e-05	1.01E-29
Sex	0.042507	0.040211. 0.044804	1.89E-284
FoFruF.NT3BLQ11-3 times a week	-0.006373	-0.011912. -0.000834	0.024141

FoFruF.NT3BLQ14-6 times a week	-0.007088	-0.012744. -0.001432	0.014041
FoFruF.NT3BLQ1Once a day	-0.008894	-0.014395. -0.003394	0.001528
FoFruF.NT3BLQ1Twice or more a day	-0.013738	-0.01942. -0.008056	2.15E-06
SYSPRS	0.11002	0.092191. 0.127848	1.25E-33
FoFruF.NT3BLQ11-3 times a week:SYSPRS	0.010954	-0.008656. 0.030564	0.273577
FoFruF.NT3BLQ14-6 times a week:SYSPRS	0.014575	-0.005434. 0.034584	0.153374
FoFruF.NT3BLQ1Once a day:SYSPRS	0.031592	0.012331. 0.050853	0.001306
FoFruF.NT3BLQ1Twice or more a day:SYSPRS	0.03848	0.018754. 0.058206	0.000132

**Consumption frequency of vegetables (FoVegF)**	**β estimate**	**95% CI**	**P-value**
(Intercept)	4.681311	4.669166. 4.693456	<2.2e–16
Age	0.002094	0.001684. 0.002505	1.52E-23
Age^2	2.30E-05	1.9e-05. 2.6e-05	4.33E-31
Sex	0.042938	0.040639. 0.045238	2.11E-289
FoVegF.NT3BLQ11-3 times a week	-0.003033	-0.010736. 0.00467	0.440287
FoVegF.NT3BLQ14-6 times a week	-0.006819	-0.014445. 0.000807	0.079684
FoVegF.NT3BLQ1Once a day	-0.007724	-0.015306. -0.000141	0.045879
FoVegF.NT3BLQ1Twice or more a day	-0.010811	-0.019346. -0.002276	0.013045
SYSPRS	0.112383	0.086051. 0.138715	6.16E-17
FoVegF.NT3BLQ11-3 times a week:SYSPRS	0.001079	-0.026701. 0.028858	0.939337
FoVegF.NT3BLQ14-6 times a week:SYSPRS	0.02299	-0.004376. 0.050356	0.099653
FoVegF.NT3BLQ1Once a day:SYSPRS	0.027237	0.000196. 0.054278	0.048360
FoVegF.NT3BLQ1Twice or more a day:SYSPRS	0.040058	0.009753. 0.070363	0.009578

**Consumption frequency of boiled potatoes (FoPotF)**	**β estimate**	**95% CI**	**P-value**
(Intercept)	4.685286	4.674373. 4.696199	<2.2e–16
Age	0.001862	0.001443. 0.002282	3.09E-18

Age^2	2.40E-05	2e-05. 2.8e-05	1.94E-33
Sex	0.04405	0.041803. 0.046296	2.73e-318
FoPotF.NT3BLQ11-3 times a week	-0.005942	-0.011453. -0.000431	0.034583
FoPotF.NT3BLQ14-6 times a week	-0.003787	-0.009282. 0.001709	0.176834
FoPotF.NT3BLQ1Once a day	0.001212	-0.004581. 0.007005	0.681726
FoPotF.NT3BLQ1Twice or more a day	-0.007603	-0.025116. 0.00991	0.394814
SYSPRS	0.109402	0.091753. 0.127052	6.49E-34
FoPotF.NT3BLQ11-3 times a week:SYSPRS	0.011192	-0.008165. 0.030549	0.257107
FoPotF.NT3BLQ14-6 times a week:SYSPRS	0.026662	0.007916. 0.045409	0.005311
FoPotF.NT3BLQ1Once a day:SYSPRS	0.036155	0.016991. 0.05532	0.000218
FoPotF.NT3BLQ1Twice or more a day:SYSPRS	0.014883	-0.043495. 0.073261	0.617290

Consumption frequency of rice/pasta (FoRicF)	β estimate	95% CI	P-value
(Intercept)	4.701528	4.690364. 4.712692	<2.2e–16
Age	0.001376	0.000952. 0.0018	2.03E-10
Age^2	2.80E-05	2.4e-05. 3.2e-05	1.66E-42
Sex	0.046233	0.043964. 0.048501	<2.2e–16
FoRicF.NT3BLQ11-3 times a week	-0.0122	-0.014791. -0.009608	2.91E-20
FoRicF.NT3BLQ14-6 times a week	-0.00815	-0.013604. -0.002696	0.003404
FoRicF.NT3BLQ1Once a day	-0.001131	-0.015953. 0.013691	0.881134
FoRicF.NT3BLQ1Twice or more a day	-0.017763	-0.05913. 0.023604	0.399997
SYSPRS	0.141646	0.13557. 0.147722	<2.2e–16
FoRicF.NT3BLQ11-3 times a week:SYSPRS	-0.011947	-0.020332. -0.003562	0.005230
FoRicF.NT3BLQ14-6 times a week:SYSPRS	-0.041799	-0.059515. -0.024082	3.77E-06
FoRicF.NT3BLQ1Once a day:SYSPRS	-0.04492	-0.097551. 0.007711	0.094366
FoRicF.NT3BLQ1Twice or more a day:SYSPRS	-0.009351	-0.134016. 0.115314	0.883117

How many glasses of the following types of beverage do you usually drink: Soda/squashes without sugar? (DriLem2Gl)	β estimate	95% CI	P-value
(Intercept)	4.688873	4.677733. 4.700013	<2.2e–16
Age	0.0017	0.001276. 0.002124	3.99E-15
Age^2	2.70E-05	2.3e-05. 3.1e-05	6.76E-41
Sex	0.046166	0.043874. 0.048458	<2.2e–16
DriLem2Gl.NT3BLQ11-6 glasses a week	-0.004701	-0.009268. -0.000133	0.043690
DriLem2Gl.NT3BLQ12-3 glasses a day	0.004936	-0.001637. 0.01151	0.141034
DriLem2Gl.NT3BLQ14 glasses or more a day	0.006264	-0.004822. 0.01735	0.268089
DriLem2Gl.NT3BLQ1Seldom/never	-0.014795	-0.019173. -0.010417	3.56E-11
SYSPRS	0.137094	0.122967. 0.15122	2.32E-80
DriLem2Gl.NT3BLQ11-6 glasses a week:SYSPRS	-0.015206	-0.031141. 0.000729	0.061443
DriLem2Gl.NT3BLQ12-3 glasses a day:SYSPRS	-0.026183	-0.049121. -0.003245	0.025272
DriLem2Gl.NT3BLQ14 glasses or more a day:SYSPRS	-0.013639	-0.052796. 0.025518	0.494793
DriLem2Gl.NT3BLQ1Seldom/never:SYSPRS	0.003605	-0.011562. 0.018773	0.641301

How often do you drink 5 glasses or more of beer, wine or spirits in one sitting? (AlcMor5GlF)	β estimate	95% CI	P-value
(Intercept)	4.668422	4.657447. 4.679398	<2.2e–16
Age	0.002091	0.00167. 0.002512	2.27E-22
Age^2	2.40E-05	2e-05. 2.7e-05	4.40E-32
Sex	0.041541	0.039163. 0.043919	8.66E-254
AlcMor5GlF.NT3BLQ1Monthly	0.011076	0.008167. 0.013986	8.66E-14
AlcMor5GlF.NT3BLQ1Weekly	0.032134	0.02543. 0.038838	5.97E-21
AlcMor5GlF.NT3BLQ1Daily	-0.020255	-0.079531. 0.039021	0.503020
SYSPRS	0.140208	0.135347. 0.145069	<2.2e–16

AlcMor5GlF.NT3BLQ1Monthly:SYSPRS	-0.02251	-0.031744. -0.013277	1.77E-06
AlcMor5GlF.NT3BLQ1Weekly:SYSPRS	-0.038632	-0.061494. -0.015771	0.000927
AlcMor5GlF.NT3BLQ1Daily:SYSPRS	0.062357	-0.098794. 0.223508	0.448199

**Serum cholesterol (SeChol)**	**β estimate**	**95% CI**	**P-value**
(Intercept)	4.653319	4.642451. 4.664187	<2.2e–16
Age	0.000859	0.000439. 0.001279	6.09E-05
Age^2	3.20E-05	2.9e-05. 3.6e-05	2.71E-60
Sex	0.045326	0.043074. 0.047578	<2.2e–16
SeChol.NT3BLM	0.010143	0.009067. 0.011219	6.28E-76
SYSPRS	0.075022	0.05484. 0.095203	3.24E-13
SeChol.NT3BLM:SYSPRS	0.010584	0.006977. 0.01419	8.90E-09

**Marital status (MaritStat)**	**β estimate**	**95% CI**	**P-value**
(Intercept)	4.661795	4.650753. 4.672837	<2.2e–16
Age	0.002943	0.002486. 0.003401	2.35E-36
Age^2	1.60E-05	1.1e-05. 2e-05	3.09E-13
Sex	0.043286	0.04101. 0.045561	4.63E-300
MaritStat.NT3BLQ1Married	-0.014232	-0.017576. -0.010887	7.62E-17
MaritStat.NT3BLQ1Widow(er)	-0.007759	-0.013393. -0.002125	0.006948
MaritStat.NT3BLQ1Divorced	-0.020521	-0.025419. -0.015623	2.23E-16
MaritStat.NT3BLQ1Separated	-0.023632	-0.033929. -0.013335	6.87E-06
SYSPRS	0.107508	0.099214. 0.115803	1.85E-141
MaritStat.NT3BLQ1Married:SYSPRS	0.034185	0.024395. 0.043976	7.81E-12
MaritStat.NT3BLQ1Widow(er):SYSPRS	0.018936	0.002673. 0.0352	0.022489
MaritStat.NT3BLQ1Divorced:SYSPRS	0.044055	0.028354. 0.059757	3.83E-08
MaritStat.NT3BLQ1Separated:SYSPRS	0.024503	-0.009408. 0.058414	0.156709

Did your parents leave each other, or get a divorce, when you were a child? (ChParDiv)	β estimate	95% CI	P-value
(Intercept)	4.68246	4.671805. 4.693115	<2.2e–16
Age	0.00178	0.001367. 0.002193	3.08E-17
Age^2	2.50E-05	2.2e-05. 2.9e-05	2.50E-38
Sex	0.04404	0.041792. 0.046288	8.96e-318
ChParDiv.NT3BLQ1Yes, before I was 7 years old	3.70E-05	-0.00639. 0.006464	0.990982
ChParDiv.NT3BLQ1Yes, when I was 7-18 years old	-0.002452	-0.00835. 0.003446	0.415130
SYSPRS	0.136114	0.131941. 0.140286	<2.2e–16
ChParDiv.NT3BLQ1Yes, before I was 7 years old:SYSPRS	-0.041559	-0.063445. -0.019673	0.000198
ChParDiv.NT3BLQ1Yes, when I was 7-18 years old:SYSPRS	-0.029027	-0.049236. -0.008819	0.004875

Estimated cardiorespiratory fitness (eCRF)	β estimate	95% CI	P-value
(Intercept)	4.988959	4.969397. 5.008522	<2.2e–16
Age	-0.001355	-0.001859. -0.00085	1.41E-07
Age^2	3.70E-05	3.3e-05. 4.2e-05	2.38E-56
Sex	0.094793	0.091076. 0.098509	<2.2e–16
eCRF	-0.005556	-0.005846. -0.005266	7.70E-303
SYSPRS	0.207941	0.186617. 0.229265	5.49E-81
eCRF:SYSPRS	-0.001933	-0.002507. -0.001359	4.26E-11

SYSPRS, polygenic risk score for systolic blood pressure.

Table S5. Interaction models for diastolic blood pressure

Serum alkaline phosphatase (SeALP)	β estimate	95% CI	P-value
(Intercept)	3.795763	3.783477. 3.80805	<2.2e–16
Age	0.014474	0.014019. 0.014929	<2.2e–16
Age^2	-0.000107	-0.000111. -0.000103	<2.2e–16
Sex	0.070511	0.067998. 0.073023	<2.2e–16
SeALP.NT3BLM	0.000429	0.000375. 0.000484	1.18E-53
DIAPRS	0.134755	0.12435. 0.145159	2.97E-141
SeALP.NT3BLM:DIAPRS	-0.000327	-0.000464. -0.00019	2.82E-06

Serum creatinine (SeCrea)	β estimate	95% CI	P-value
(Intercept)	3.81759	3.804328. 3.830853	<2.2e–16
Age	0.014392	0.013934. 0.014849	<2.2e–16
Age^2	-0.000106	-0.00011. -0.000102	<2.2e–16
Sex	0.070722	0.067958. 0.073485	<2.2e–16
SeCrea.NT3BLM	0.000113	3.5e-05. 0.000191	0.004631
DIAPRS	0.157112	0.141897. 0.172327	1.05E-90
SeCrea.NT3BLM:DIAPRS	-0.000557	-0.000738. -0.000376	1.76E-09

Marital status (MaritStat)	β estimate	95% CI	P-value
(Intercept)	3.813276	3.800876. 3.825676	<2.2e–16
Age	0.015146	0.014632. 0.01566	<2.2e–16
Age^2	-0.000112	-0.000116. -0.000107	<2.2e–16
Sex	0.071138	0.068583. 0.073692	<2.2e–16
MaritStat.NT3BLQ1Married	-0.012418	-0.016193. -0.008642	1.15E-10

MaritStat.NT3BLQ1Widow(er)	-0.007405	-0.013768. -0.001042	0.022554
MaritStat.NT3BLQ1Divorced	-0.012946	-0.018479. -0.007413	4.53E-06
MaritStat.NT3BLQ1Separated	-0.017024	-0.028569. -0.00548	0.003850
DIAPRS	0.103129	0.096276. 0.109983	1.55E-189
MaritStat.NT3BLQ1Married:DIAPRS	0.012967	0.004878. 0.021055	0.001678
MaritStat.NT3BLQ1Widow(er):DIAPRS	-0.011578	-0.025108. 0.001952	0.093498
MaritStat.NT3BLQ1Divorced:DIAPRS	0.020318	0.007264. 0.033372	0.002284
MaritStat.NT3BLQ1Separated:DIAPRS	-0.003206	-0.031164. 0.024752	0.822169

**Do you use, or have you used snus? (SnuEvEd)**	**β estimate**	**95% CI**	**P-value**
(Intercept)	3.826741	3.814819. 3.838663	<2.2e–16
Age	0.014333	0.013868. 0.014799	<2.2e–16
Age^2	-0.000105	-0.00011. -0.000101	<2.2e–16
Sex	0.072215	0.069506. 0.074924	<2.2e–16
SnuEvEd.NT3BLQ1Yes	0.003201	-0.000687. 0.007089	0.106577
DIAPRS	0.11563	0.111981. 0.119278	<2.2e–16
SnuEvEd.NT3BLQ1Yes:DIAPRS	-0.023321	-0.032511. -0.014131	6.59E-07

DIAPRS, polygenic risk score for diastolic blood pressure.

Supplemental plots

PRS_{SBP} × environment interaction effects on systolic blood pressure

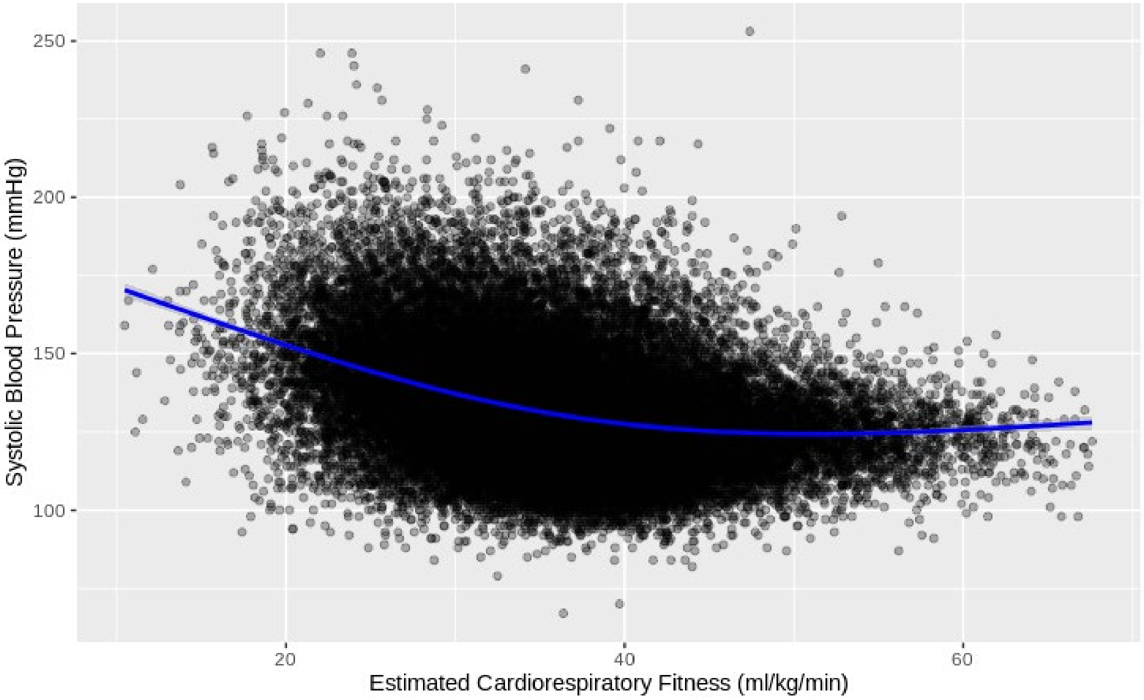

Figure S1. Association between estimated cardiorespiratory fitness on systolic blood pressure

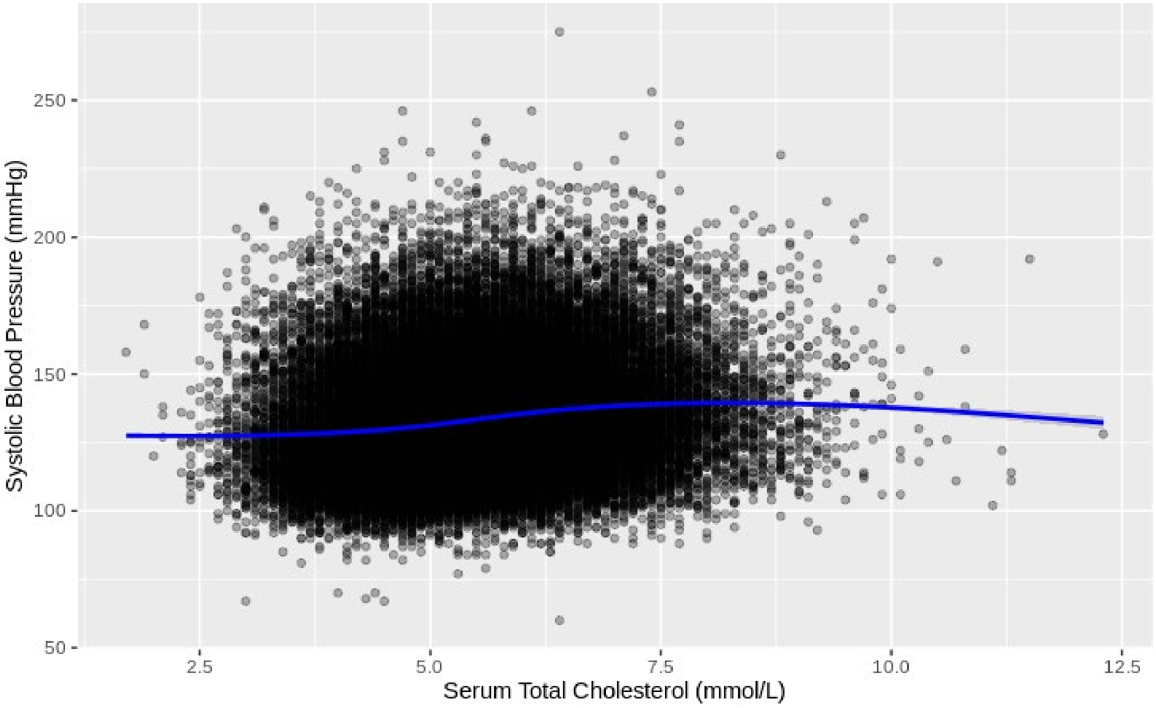

Figure S2. Association between total cholesterol on systolic blood pressure

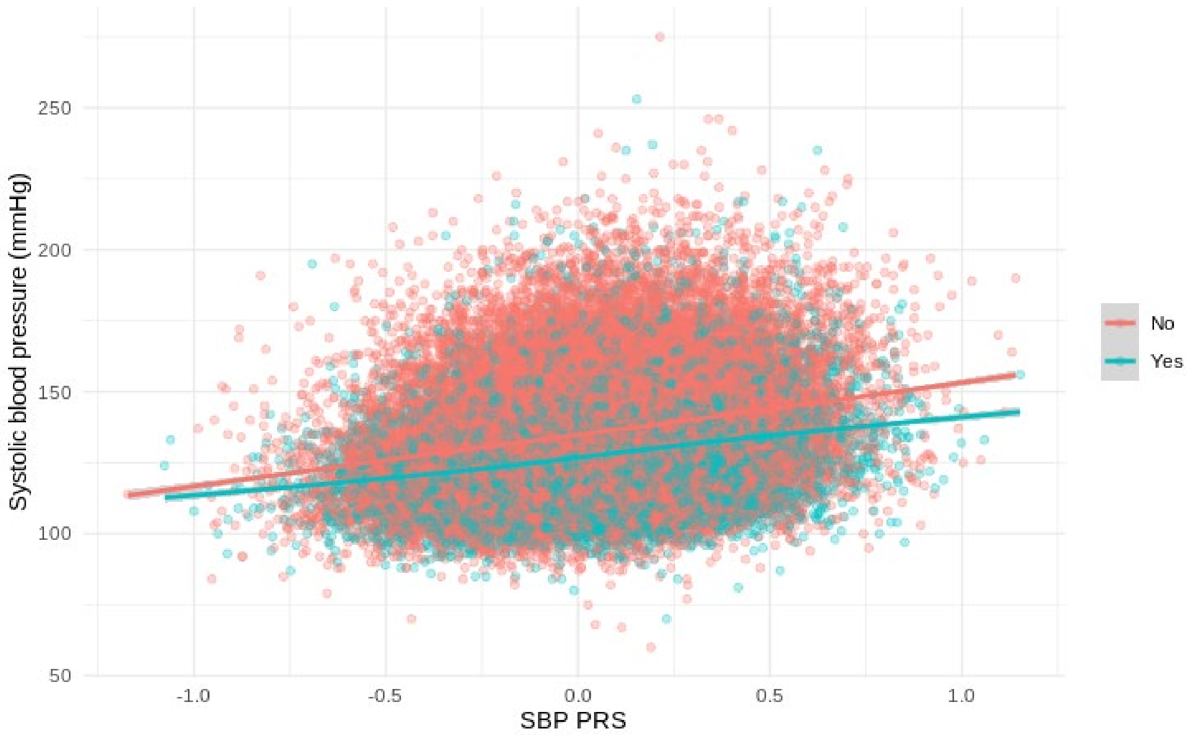

Figure S3. PRS_{SBP} × "Did your mother smoke when you were growing up?"

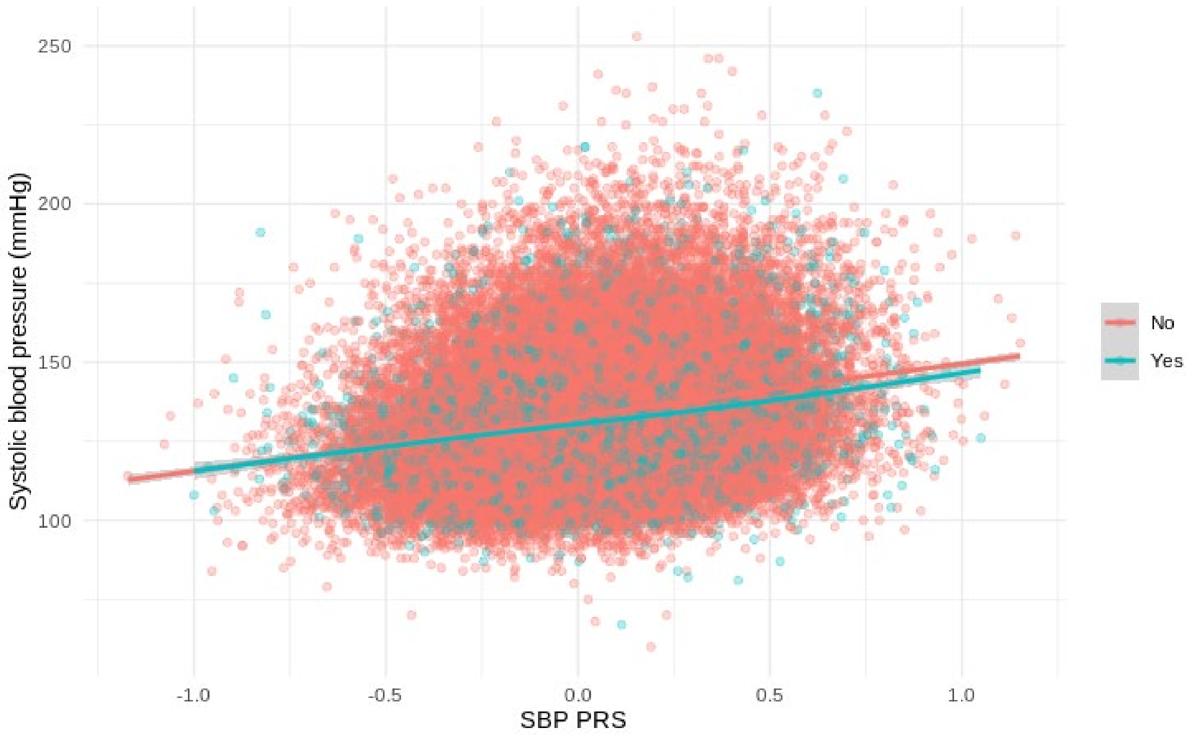

Figure S4. PRS_{SBP} × "Do you use, or have you used snus?"

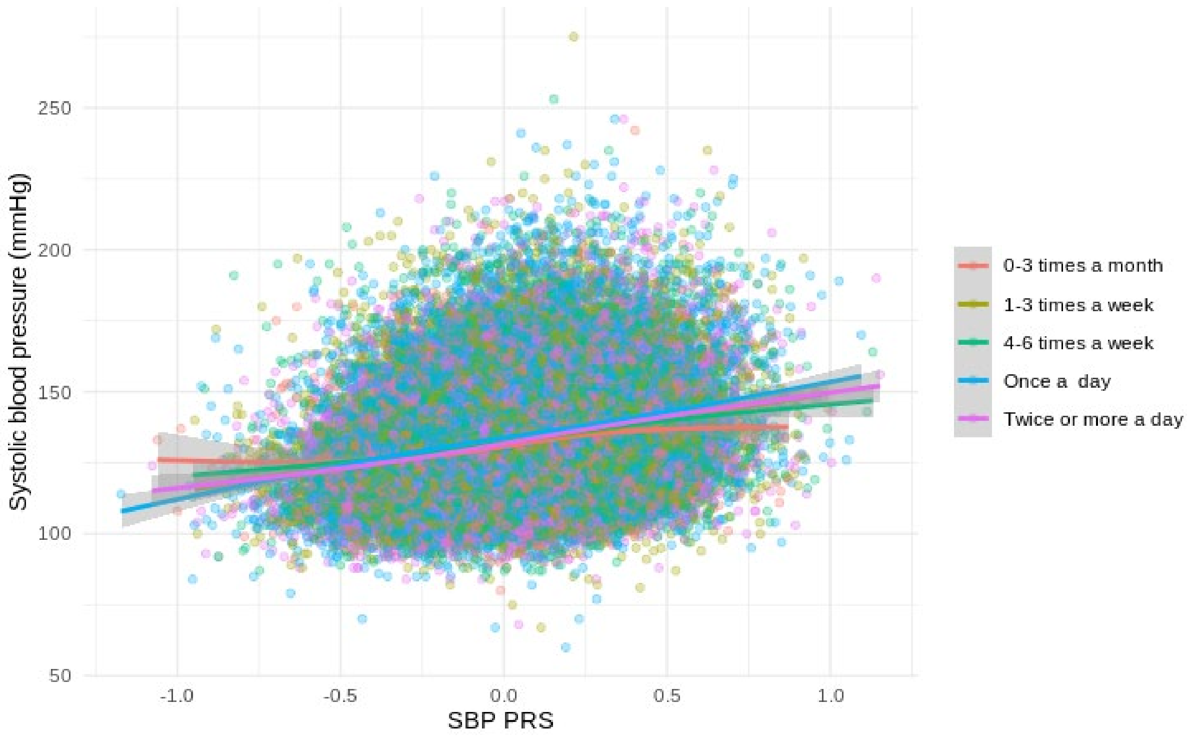

Figure S5. PRS_{SBP} × Consumption frequency of fruit and berries

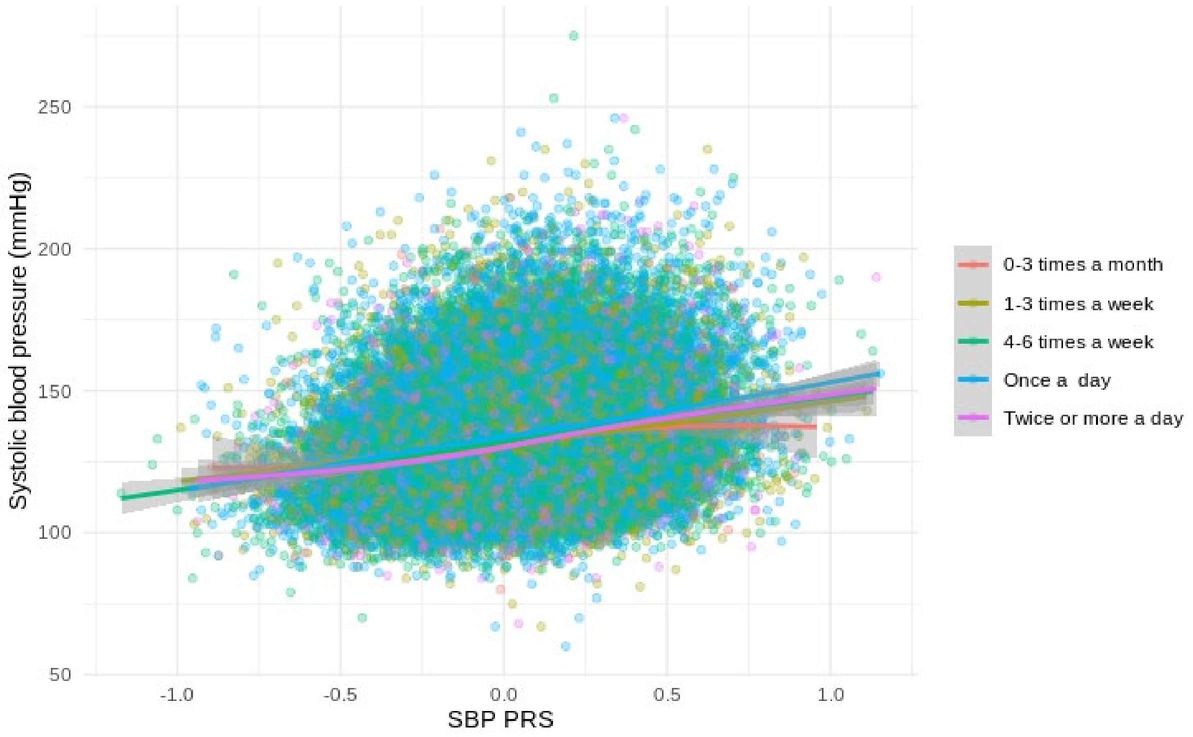

Figure S6. PRS_{SBP} × Consumption frequency of vegetables

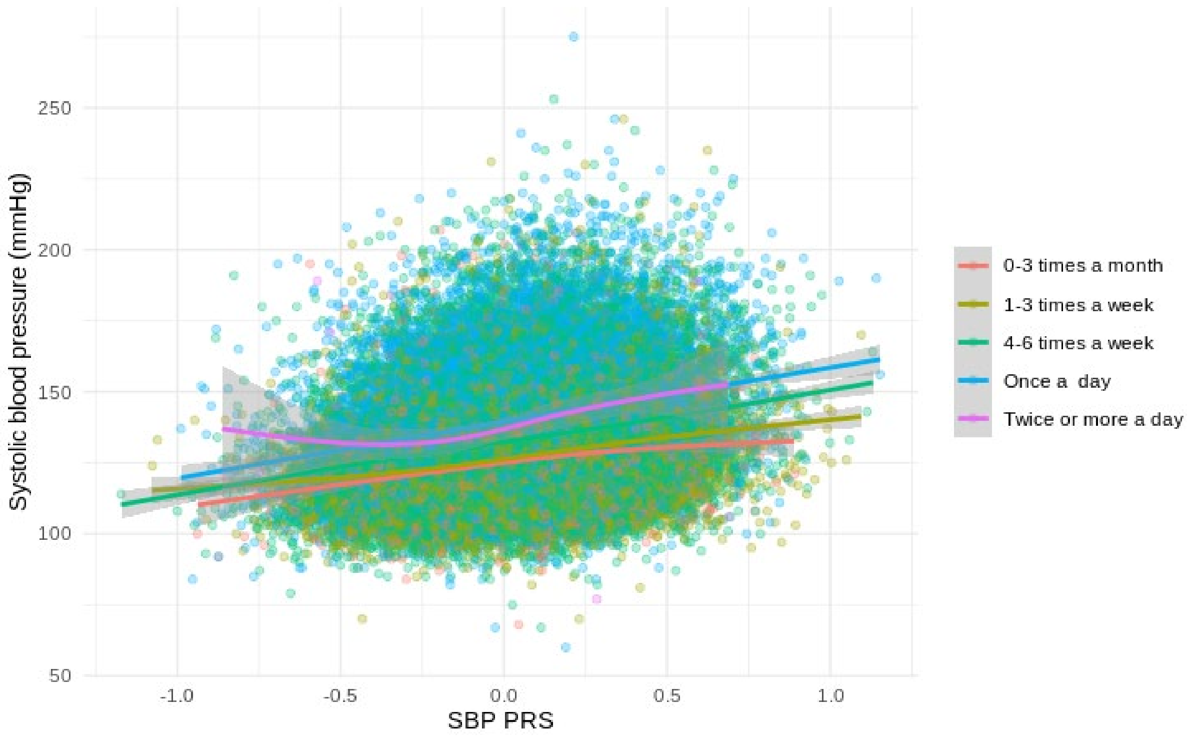

Figure S7. PRS_{SBP} × Consumption frequency of boiled potatoes

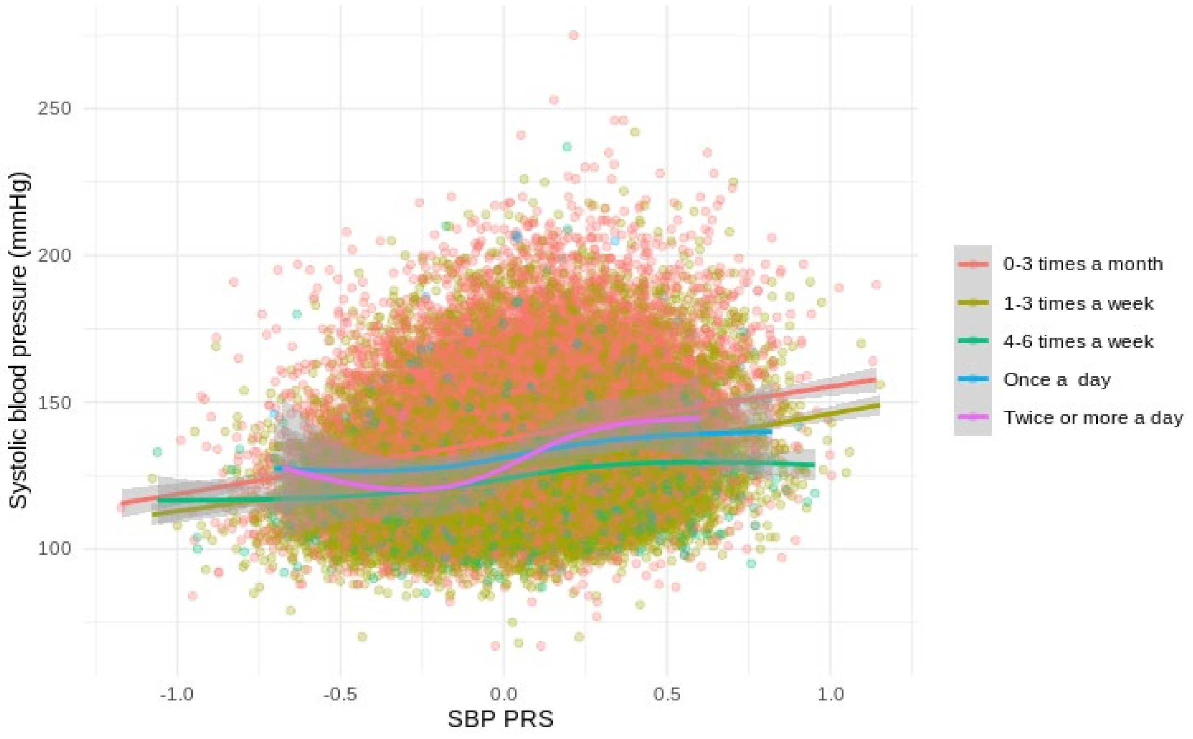

Figure S8. PRS_{SBP} × Consumption frequency of rice/pasta

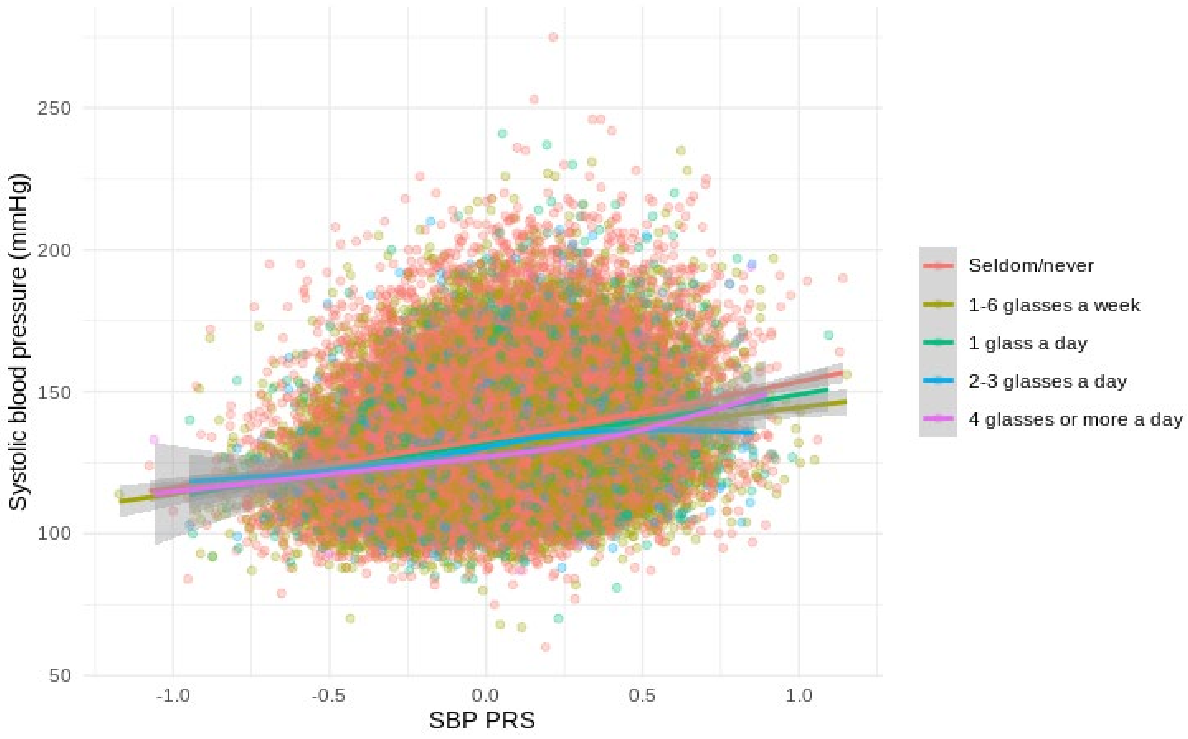

Figure S9. PRS_{SBP} × Consumption frequency of sugar-free soda/squashes

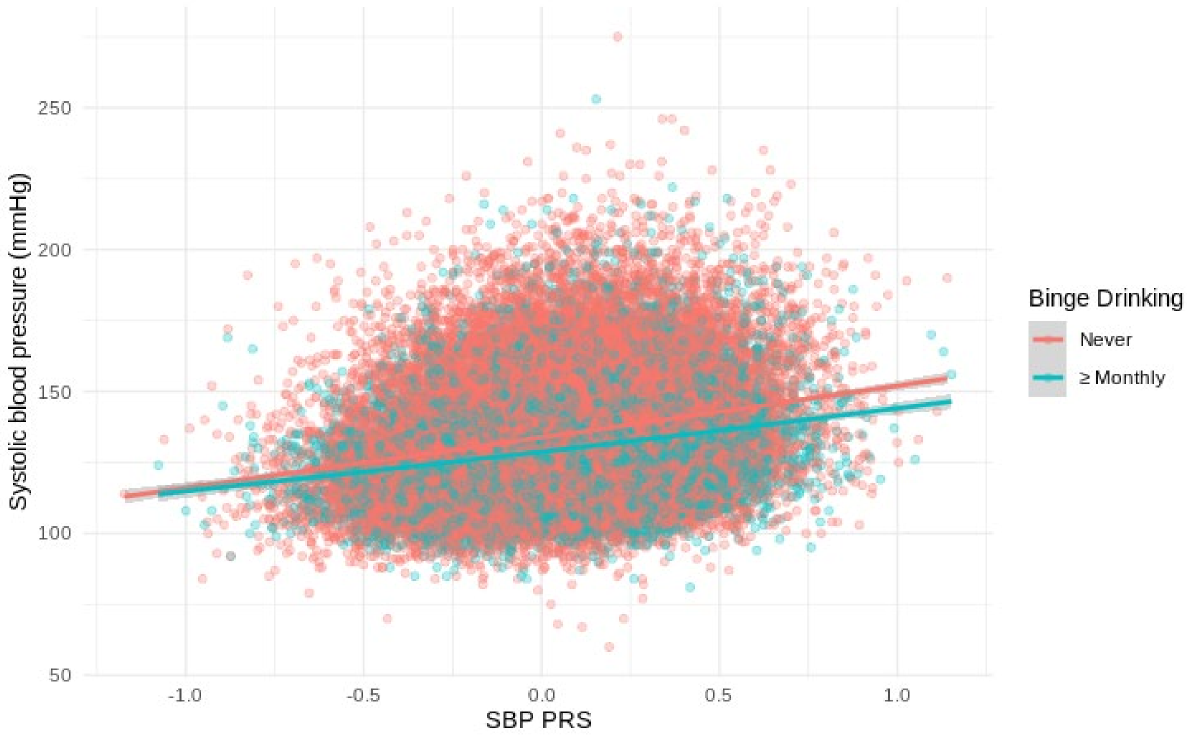

Figure S10. PRS_{SBP} × "How often do you drink 5 glasses or more of beer, wine or spirits in one sitting?"

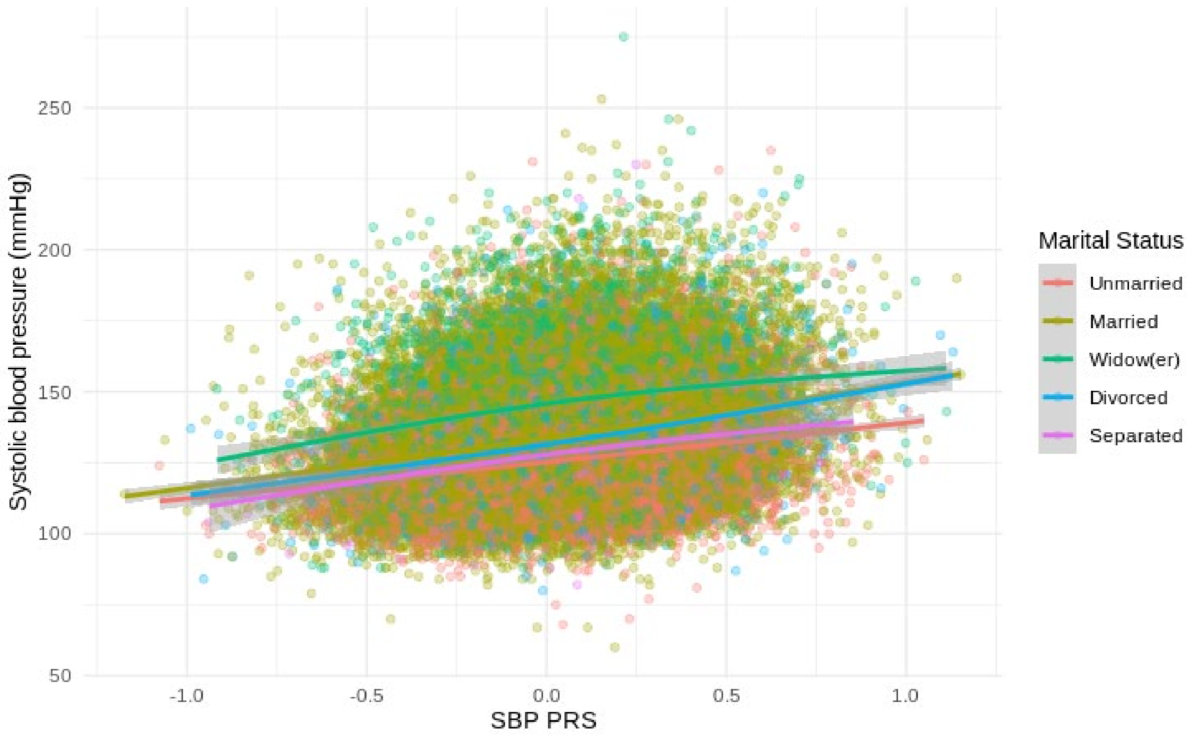

Figure S11. PRS_{SBP} × Marital status

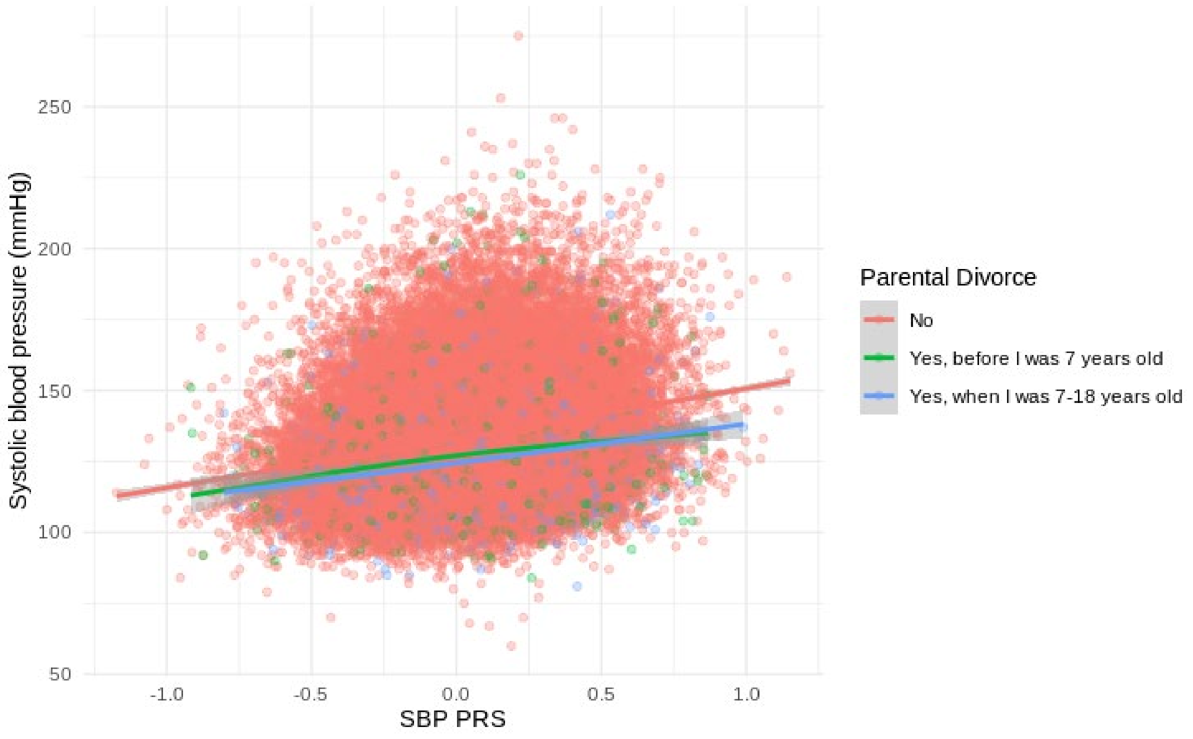

Figure S12. PRS_{SBP} × "Did your parents leave each other, or get a divorce, when you were a child?"

PRS_{DBP} × environment interaction effects on diastolic blood pressure

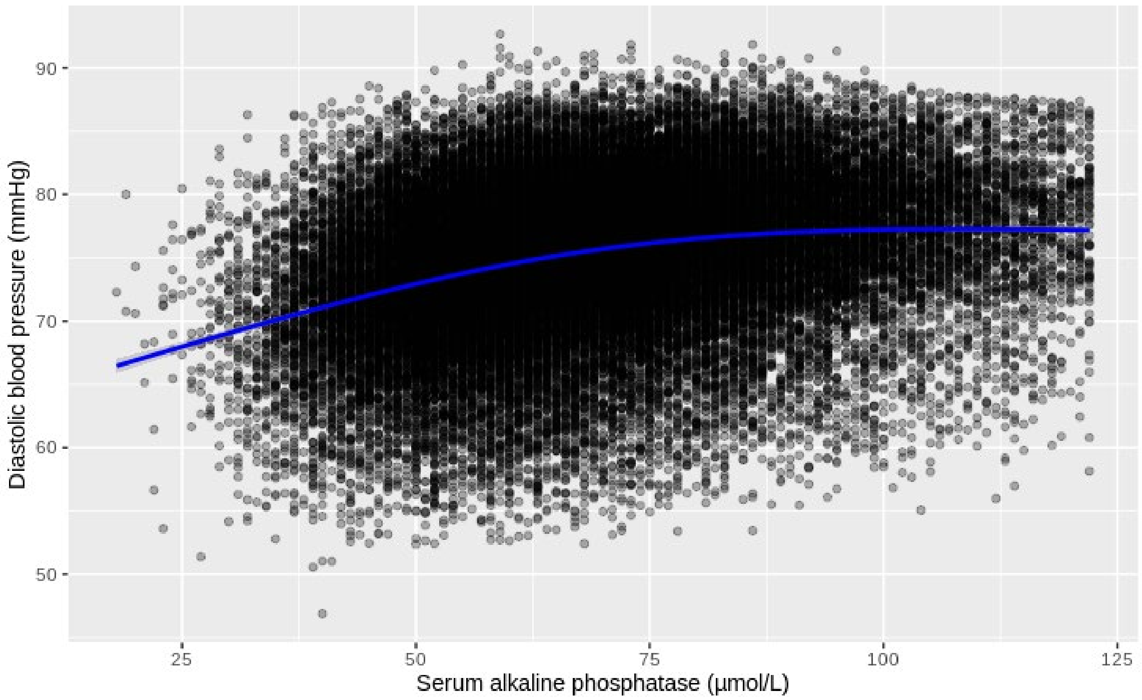

Figure S13. Association between serum alkaline phosphatase and diastolic blood pressure

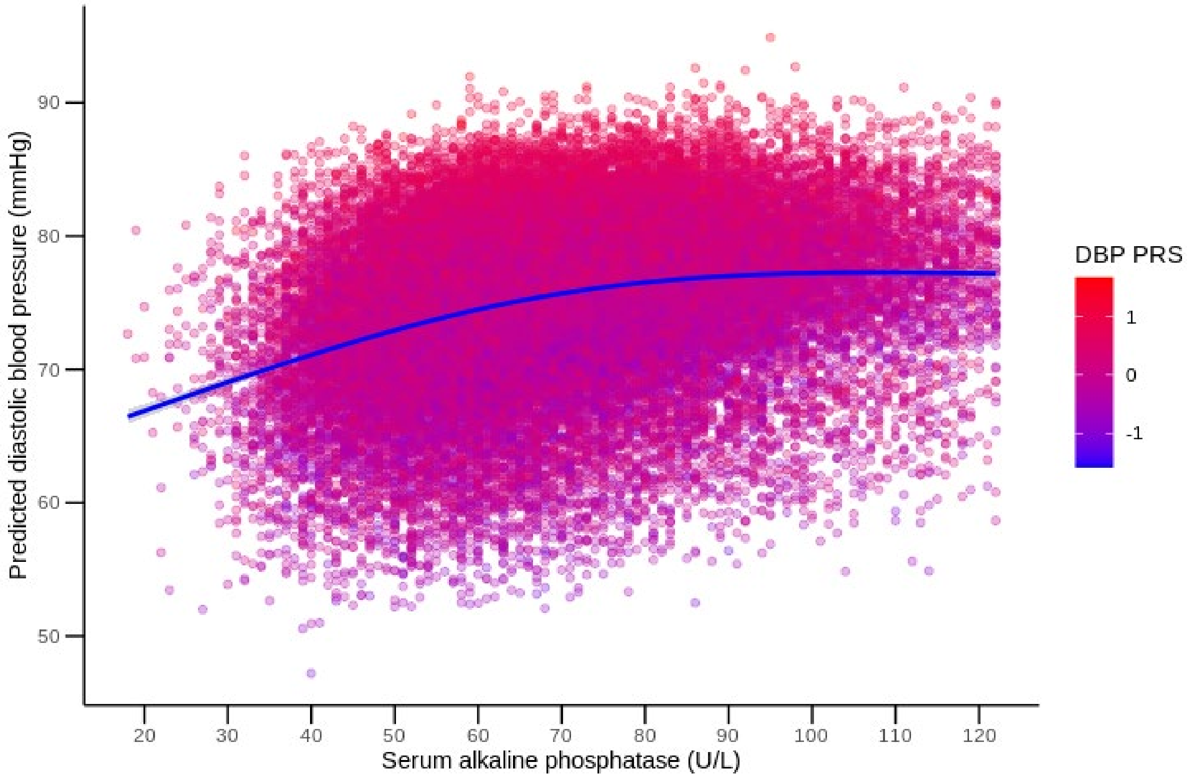

Figure S14. PRS_{DBP} × Serum alkaline phosphatase

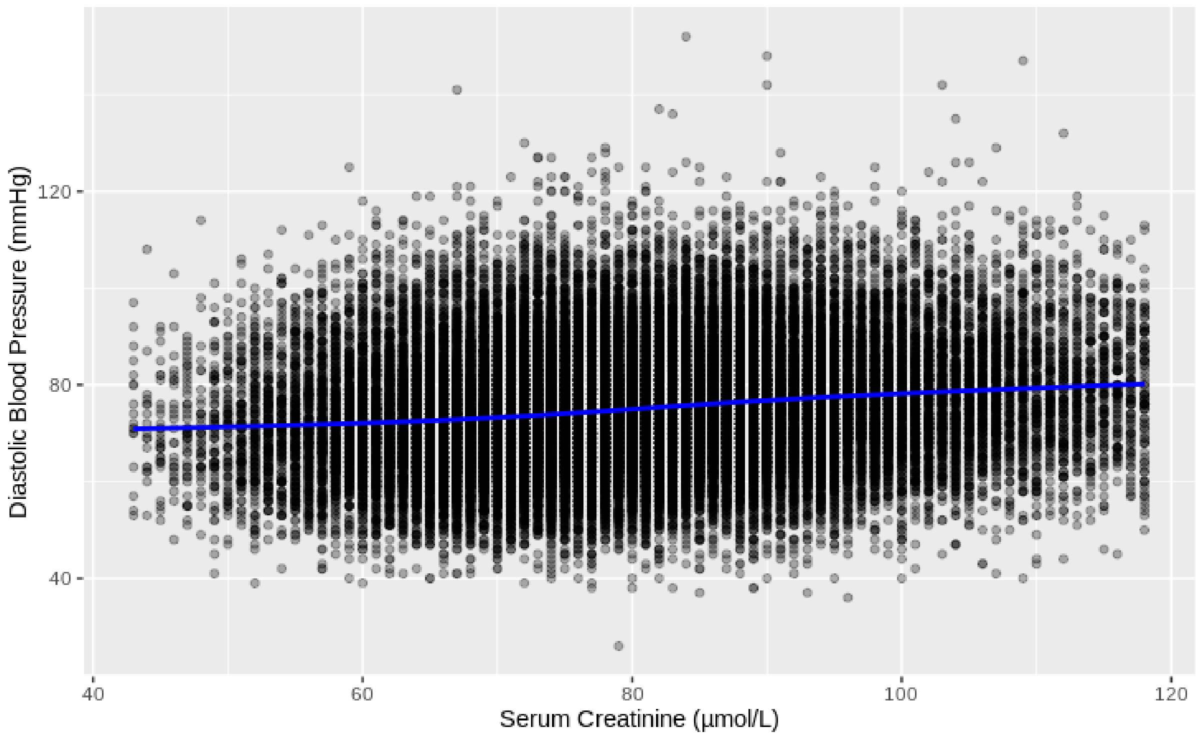

Figure S15. Association between serum creatinine and diastolic blood pressure

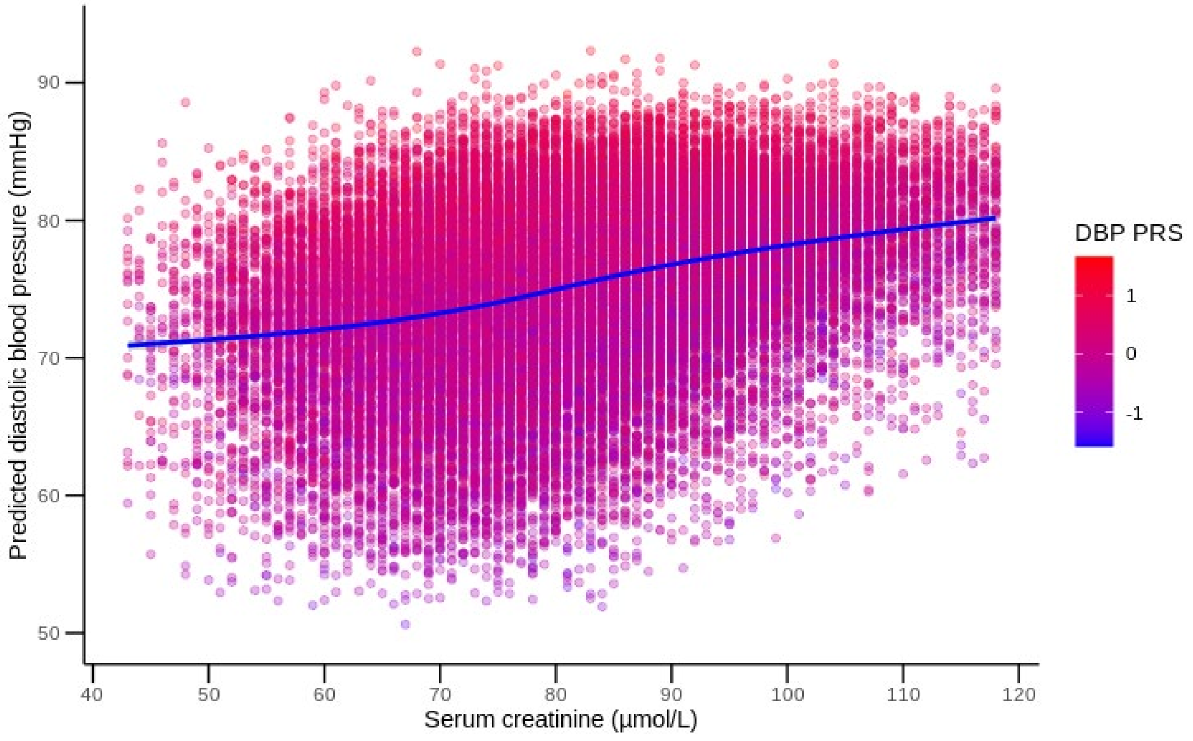

Figure S16. PRS_{DBP} × Serum creatinine

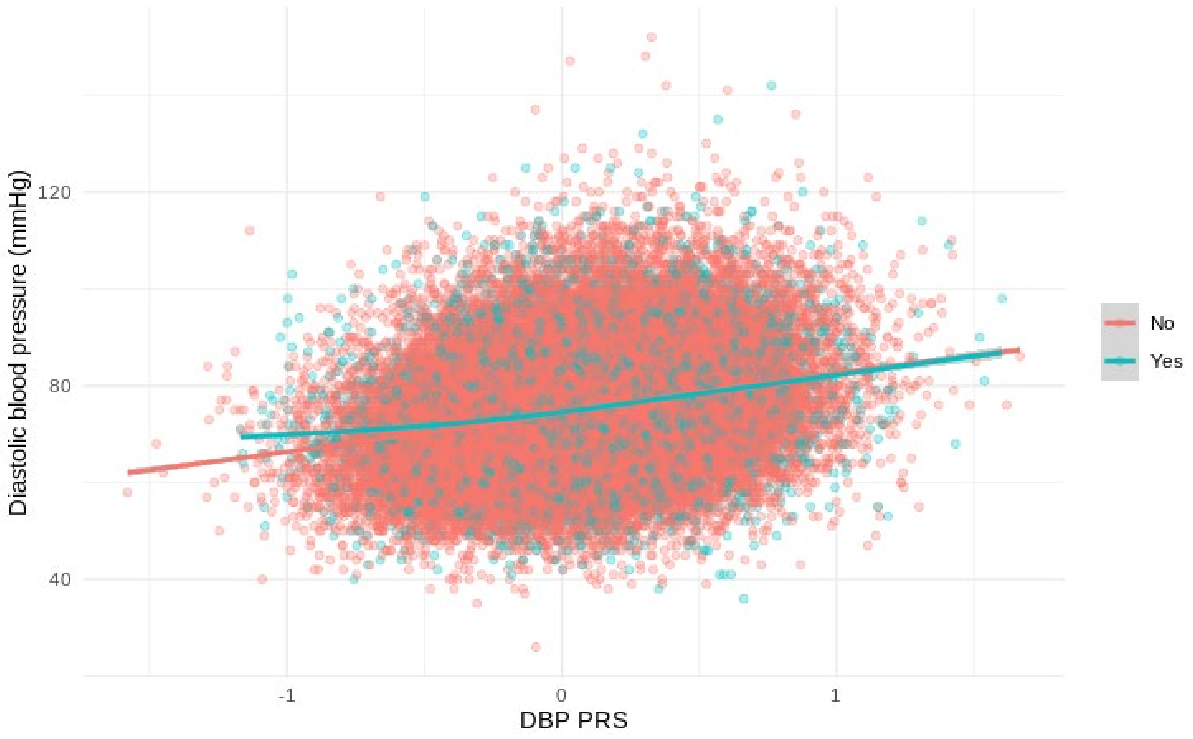

Figure S17. PRS_{DBP} × "Do you use, or have you used snus?"

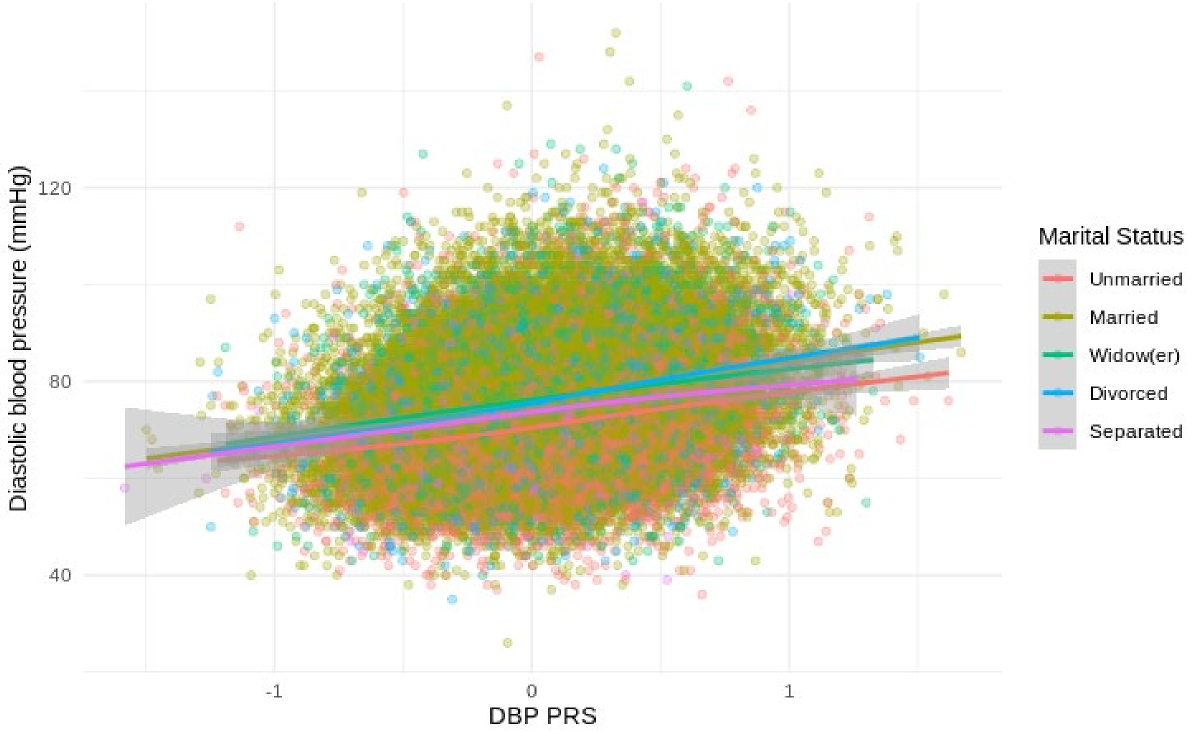

Figure S18. PRS_{DBP} × Marital status
